# Vaccination of people with solid tumors and diabetes: existing evidence and recommendations. A position statement from a multidisciplinary panel of scientific societies

**DOI:** 10.1007/s40618-025-02586-5

**Published:** 2025-04-23

**Authors:** Marco Gallo, Angioletta Lasagna, Valerio Renzelli, Lelio Morviducci, Alessio Cortellini, Matteo Monami, Giampiero Marino, Stefania Gori, Matteo Verzé, Alberto Ragni, Enzo Tuveri, Laura Sciacca, Stella D’Oronzo, Dario Giuffrida, Annalisa Natalicchio, Francesco Giorgino, Nicola Marrano, Maria Chiara Zatelli, Monica Montagnani, Francesco Felicetti, Rossella Mazzilli, Stefano Fogli, Tindara Franchina, Antonella Argentiero, Riccardo Candido, Francesco Perrone, Gianluca Aimaretti, Angelo Avogaro, Nicola Silvestris, Antongiulio Faggiano

**Affiliations:** 1 Endocrinology and Metabolic Diseases Unit, Azienda Ospedaliero-Universitaria SS Antonio e Biagio e Cesare Arrigo of Alessandria, Alessandria, 15121 Italy; 2 Medical Oncology, Fondazione IRCCS Policlinico San Matteo, 27100 Pavia, Italy; 3 Diabetologist and Endocrinologist, Italian Association of Clinical Diabetologists, Rome, Italy; 4 Diabetology and Nutrition Unit, Department of Medical Specialties, ASL Roma 1– S. Spirito Hospital, Rome, Italy; 5 Operative Research Unit of Medical Oncology, Fondazione Policlinico Universitario Campus Bio-Medico, Roma, Italy; 6 Department of Medicine and Surgery, Università Campus Bio-Medico di Roma, Roma, Italy; 7 Department of Surgery and Cancer, Hammersmith Hospital Campus, Imperial College London, London, UK; 8 Diabetology, Careggi Hospital and University of Florence, Florence, Italy; 9 Internal Medicine Department, Ospedale dei Castelli, Asl Roma 6, Ariccia, RM Italy; 10 Medical Oncology, IRCCS Sacro Cuore Don Calabria Hospital, Negrar di Valpolicella, Verona, Italy; 11 Diabetology, Endocrinology and Metabolic Diseases Unit, ASL-Sulcis, Carbonia, Italy; 12 Department of Clinical and Experimental Medicine, Endocrinology Section, University of Catania Catania, Catania, Italy; 13 Oncology and Oncohematology Division, Acquaviva delle Fonti; and Medicine and Surgery Department, “F. Miulli” General Regional Hospital, LUM University, Casamassima, Bari, Italy; 14 Department of Oncology, Istituto Oncologico del Mediterraneo, Viagrande, Catania Italy; 15 Department of Precision and Regenerative Medicine and Ionian Area, Section of Internal Medicine, Endocrinology, Andrology and Metabolic Diseases, University of Bari Aldo Moro, Bari, Italy; 16 Section of Endocrinology, Geriatrics and Internal Medicine, Department of Medical Sciences, University of Ferrara, Ferrara, Italy; 17 Department of Precision and Regenerative Medicine and Ionian Area, Section of Pharmacology, University of Bari Aldo Moro, Bari, Italy; 18 Division of Oncological Endocrinology, Department of Oncology, University Hospital A.O.U. “Città della Salute e della Scienza di Torino”, Torino, 10126 Italy; 19 Endocrinology Unit, Department of Clinical & Molecular Medicine, Sant’Andrea Hospital, Sapienza University of Rome, Rome, Italy; 20 Clinical Pharmacology and Pharmacogenetics Unit, Department of Clinical and Experimental Medicine, University of Pisa, Pisa, Italy; 21 Medical Oncology Unit, Department of Human Pathology “G. Barresi”, University of Messina, Messina, Italy; 22 Medical Oncology Department, IRCCS Istituto “Tumori Giovanni Paolo II”, Bari, Italy; 23 Department of Medical Surgical and Health Sciences, University of Trieste, Trieste, 34149 Italy; 24 Clinical Trials Unit, National Cancer Institute, Naples, Italy; 25 Endocrinology, Department of Translational Medicine, Università del Piemonte Orientale, Novara, Italy; 26 Fondazione Diabete Ricerca SID, Rome, Italy

**Keywords:** Diabetes, Cancer, Vaccines, Immunocompromised, COVID-19, Influenza, Pneumonitis, Vaccine hesitancy, Vaccine-preventable diseases (VPDs)

## Abstract

Diabetes and cancer are two of the most common public health concerns worldwide. The complex interplay of these two conditions is a growing area of research, as patients with diabetes are at increased risk for developing cancer, and vice versa. Furthermore, both patient populations show increased risk of many communicable infectious diseases and their adverse consequences, while vaccination can play a crucial role in their prevention, improving patient outcomes. Vaccination should represent a standard part of care for patients with cancer, diabetes, and both the diseases simultaneously, including people undergoing cancer treatment or in remission. Several international guidelines provide recommendations for vaccinating people with cancer or diabetes, but the two conditions have not been specifically evaluated together. Here we present a multidisciplinary consensus position paper on vaccination in patients with cancer and diabetes. The position paper is the result of a collaborative effort between experts from the Italian Association of Medical Oncology (AIOM), Italian Association of Medical Diabetologists (AMD), Italian Society of Diabetology (SID), Italian Society of Endocrinology (SIE), and Italian Society of Pharmacology (SIF). The paper provides a comprehensive overview of the current state-of-the-art knowledge on vaccination in patients with cancer and diabetes. It discusses the importance of vaccination in preventing infections, focuses attention on the need to consider the unique challenges faced by patients with cancer and diabetes when it comes to vaccine administration, and highlights the need for coordinated care to optimize treatment outcomes. Overall, the consensus position paper provides healthcare professionals caring for patients with cancer and diabetes recommendations on the use of various vaccines, including influenza, COVID-19, HZV, and HPV vaccines, as well as guidance on how to address common concerns and challenges related to vaccine administration.

**Supplementary Information:**

The online version contains supplementary material available at 10.1007/s40618-025-02586-5.

## Introduction

The very recent COVID-19 pandemic, together with the strategies implemented for tackling its spread, have highlighted the importance of vaccination strategies for immunization from communicable diseases. Immunization from vaccines has always been considered a very effective and safe tool for the prevention of infectious diseases and for prolonging lifespan: to date, some communicable diseases have been completely eradicated thanks to vaccines, while others no longer represent a public health issue, at least in the developed world [1].

**Fig. 1:**
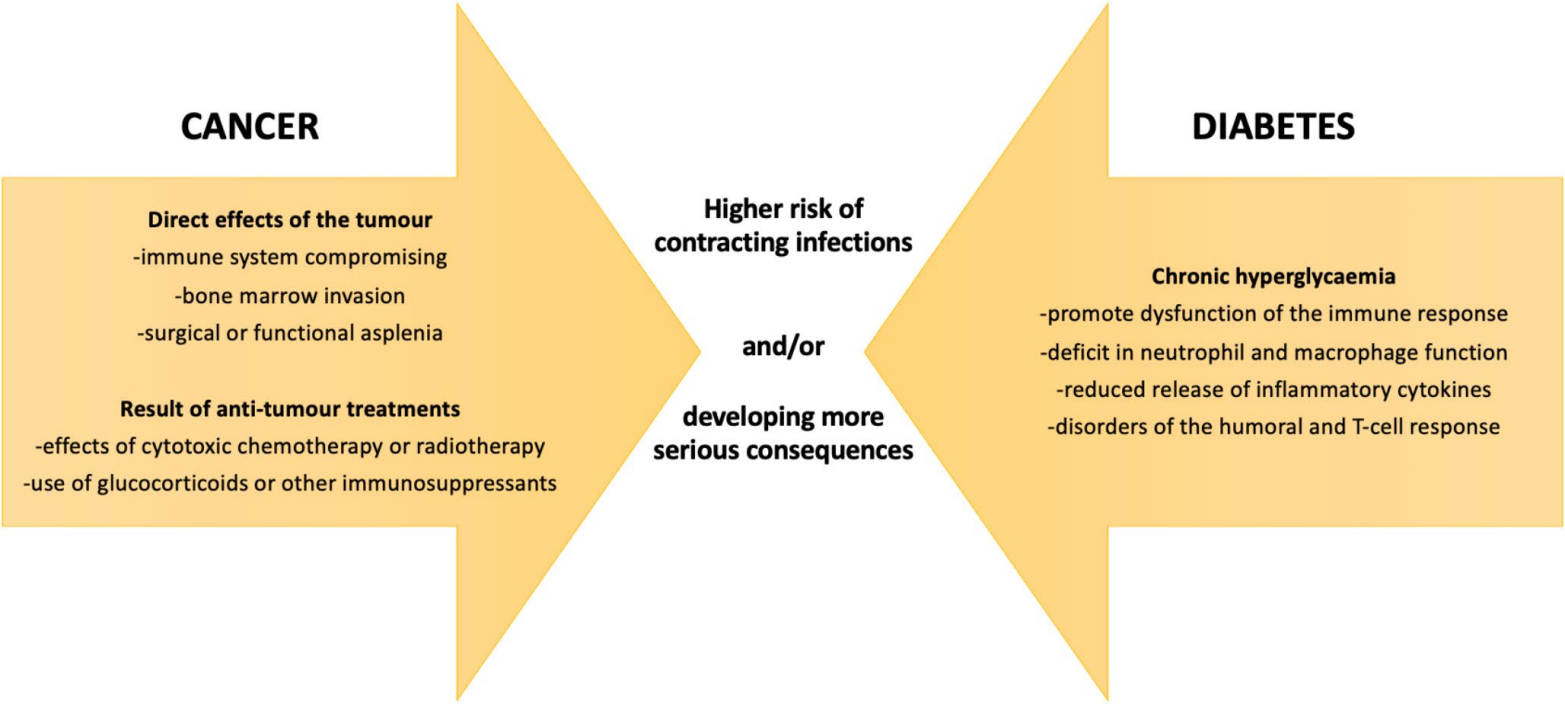
Mechanisms by which cancer and diabetes can increase the risk of contracting infections or developing more serious consequences

Compared to the general population, several groups of individuals present a specific risk of contracting infections or developing more serious consequences. Definitively, cancer patients and people with diabetes are at increased risk from these complications, due to their overall frailty and their state of relative immunosuppression (Fig. 1).

Oncological patients are particularly susceptible to infections and vulnerable to their consequences: this is due to the direct effects of the tumour (e.g., immune system compromission, bone marrow invasion, surgical or functional asplenia) and/or as a result of anti-tumour treatments (effects of cytotoxic chemotherapy or radiotherapy, use of glucocorticoids, or other immunosuppressants) [2, 3]. The most relevant oncologic scientific societies have published recommendations on vaccination against vaccine-preventable diseases in cancer patients beyond those for cancer-causing viruses [4–6].

Subjects with diabetes are also more vulnerable to infectious diseases (e.g., skin and soft tissue infections, pneumonia, urinary tract infections, and sepsis) compared to the general population, with a higher risk of hospitalization, longer hospital stay, and severe complications [7–10]. From a pathophysiological standpoint, chronic hyperglycaemia is thought to promote dysfunction of the immune response primarily through a deficit in neutrophil and macrophage function, reduced release of inflammatory cytokines, and disorders of the humoral and T-cell response, failing to overcome the spread of invading pathogens [11]. Accordingly, the Italian national vaccination plan, as well as several programs from other countries, include patients with diabetes in the “at risk” categories for which additional vaccinations are recommended, compared to the general population [12]. The American Diabetes Association (ADA) Standard of Care for diabetes, as well as many other international diabetes guidelines, recommends to provide routinely endorsed vaccinations for children and adults with diabetes as indicated by age [13, 14].

Diabetes is also the most common comorbidity in patients with cancer [15]. Consequently, in the not unusual event of subjects simultaneously affected by diabetes and cancer, the coexistence of the two conditions poses an even more important challenge in the prevention through vaccination of communicable diseases and their potential life-threatening consequences, often capable of nullifying the great results attainable with current anti-tumour treatments. Moreover, the response to some vaccinations in these subjects could be weaker than healthy individuals of the same age: a limitation that can be overcome by reducing the spread of the infective agent, through greater implementation of immunization of both the patients themselves and their caregivers [16]. Consequently, there is a growing need to implement suitable vaccination strategies, through campaigns that must integrate the different healthcare providers and specialties.

The knowledge of this ever-evolving issue, especially if applied to special populations such as patients with diabetes, cancer, or both the diseases, allows Diabetologists and Oncologists (as well as General Practitioners, Internal Medicine specialists, Infective Diseases specialists, and several other healthcare figures) to safeguard the undisputed health and social value of this fundamental preventive strategy.

The purpose of this position statement from a multidisciplinary panel of experts from different Italian scientific societies (AIOM, Italian Association of Medical Oncology; AMD, Italian Association of Medical Diabetologists; SID, Italian Society of Diabetology; SIE, Italian Society of Endocrinology; and SIF, Italian Society of Pharmacology) is to provide a critical overview of vaccination strategies currently recommended for people with diabetes and for cancer patients, paying particular attention to subjects simultaneously suffering from both conditions.

## General review of existing guidelines and recommendations

Both in vitro and in vivo studies have shown that diabetes confers an increased susceptibility risk of developing infectious diseases and a higher hazard of death due to more severe course of them. Therefore, besides the microvascular and macrovascular complications of diabetes, infectious diseases must also be taken into highest consideration. Multiple mechanisms, secondary to chronic hyperglycemia, are implicated in the higher frequency and severity of infections in patients with diabetes. Similarly, in patients suffering from malignant tumors, the immune system is compromised due to multiple issues including chronic inflammation, impairment of the hematopoietic system as well as treatments that compromise immune function. Therefore, vaccine-preventable diseases must necessarily represent a focus for oncologists to undertake appropriate interventions in cancer patients.

The Centers for Disease Control and Prevention (CDC) Advisory Committee on Immunization Practices (ACIP) provides vaccination schedules specifically for children, adolescents, and adults, and recommends influenza and pneumococcal vaccines for all individuals with diabetes along with Hepatitis B, tetanus, diphtheria, pertussis, meningococcal disease, respiratory syncytial virus, COVID-19 and Herpes Zoster. The ACIP evidence review has evolved over the years and, in 2010, the Grading of Recommendations Assessment, Development and Evaluation (GRADE) system was adopted, subsequently arriving in 2018 at the Evidence to Decision or Evidence to Recommendation frameworks with the aim of helping to use evidence in a structured and transparent way to make decisions regarding clinical recommendations [17].

The ADA Standards of Medical Care in Diabetes has adopted the same recommendations as the ACIP [14]. Similarly, the 2022 American Association of Clinical Endocrinology (AACE) Diabetes Guideline supports the recommendations of the CDC ACIP, providing, in a paramount section (Question 28), 11 recommendations and the evidence base for each statement on the types of vaccines adults with diabetes should receive [18].

In Italy, in 2018, a consensus statement has been signed by diabetes scientific societies (AMD and SID), General Practitioners associations (FIMMG and SIMG), and Hygienist scientific society (SItI), in order to provide a Recommended Vaccinations Guideline in adult patients with diabetes. The impact and benefits of vaccinations against influenza, pneumococcal, meningococcal, diphtheria-tetanus-pertussis, COVID-19, hepatitis B, mumps-pertussis-rubella (MPS) and Zoster have been traced. The ultimate aim is to increase vaccination coverage which is still far from safety standards [19].

With similar goals, the American Society of Clinical Oncology (ASCO) has stepped into a five-year cooperative agreement with the CDC, supporting guideline development and efforts in providers’ and patients’ education with the final aim of increasing vaccination rates among cancer patients. For all seasonal vaccines as well as age- and risk-based vaccines (influenza, respiratory syncytial virus, COVID-19, tetanus, diphtheria, pertussis, hepatitis B, Zoster, pneumococcal vaccines, Human Papilloma Virus-HPV), vaccination status of the patient should be settled up, and vaccination should be performed 2–4 weeks before any scheduled cancer treatment and taking into account between live or non-live virus vaccines, too [4].

In a recent paper, the Italian Association of Medical Oncology (AIOM) has strongly reinforced the recommendations on seasonal influenza, pneumococcal infection and SARS-CoV-2 vaccinations, underlining the deleterious effects of vaccine hesitancy in patients with malignancies [3].

## Vaccinations in cancer patients

### Specific challenges of vaccinating patients with cancer

It is important to provide cancer patients with direct protection against infectious agents through immunization, and oncologists should provide adequate counselling and encourage adherence to vaccination. However, considering that the cancer patient’s immune system, due to the state of immunosuppression, may respond sub-optimally to vaccination, it is necessary to consider vaccinating also people who are in close contact, such as cohabitants, caregivers, and the healthcare personnel in charge of the patient (doctors, nurses, social and healthcare workers). Close family members or persons in close contact with the patient should be assessed for their actual vaccination status and possibly be (re)vaccinated [20].

ASCO recommends vaccinating all family members and close contacts, if possible [4]. The Infectious Diseases Society of America (IDSA) 2013 guidelines state that immunocompetent individuals living in a household with immunocompromised patients can safely receive all recommended live and non-live vaccines, with some exceptions and precautions [21]. IDSA guidelines recommend against administering oral polio vaccine to contacts/family members of immunocompromised patients and against administering live attenuated influenza vaccine (LAIV vaccine administered by intranasal spray) to contacts/family members of patients who have recently received a bone marrow transplant or who develop graft versus host disease (GVHD). Vaccination against Rotavirus is strongly discouraged in family members of cancer patients [20].

The Italian Ministry of Health, for the 2024–2025 season, recommends seasonal influenza vaccination for cancer patients undergoing chemotherapy, for family members and contacts (adults and children) of cancer patients, and for physicians and health care/social care staff, since they can transmit influenza to those at high risk of influenza complications [22].

### Efficacy of vaccinations on immune response in cancer patients

**Fig. 2:**
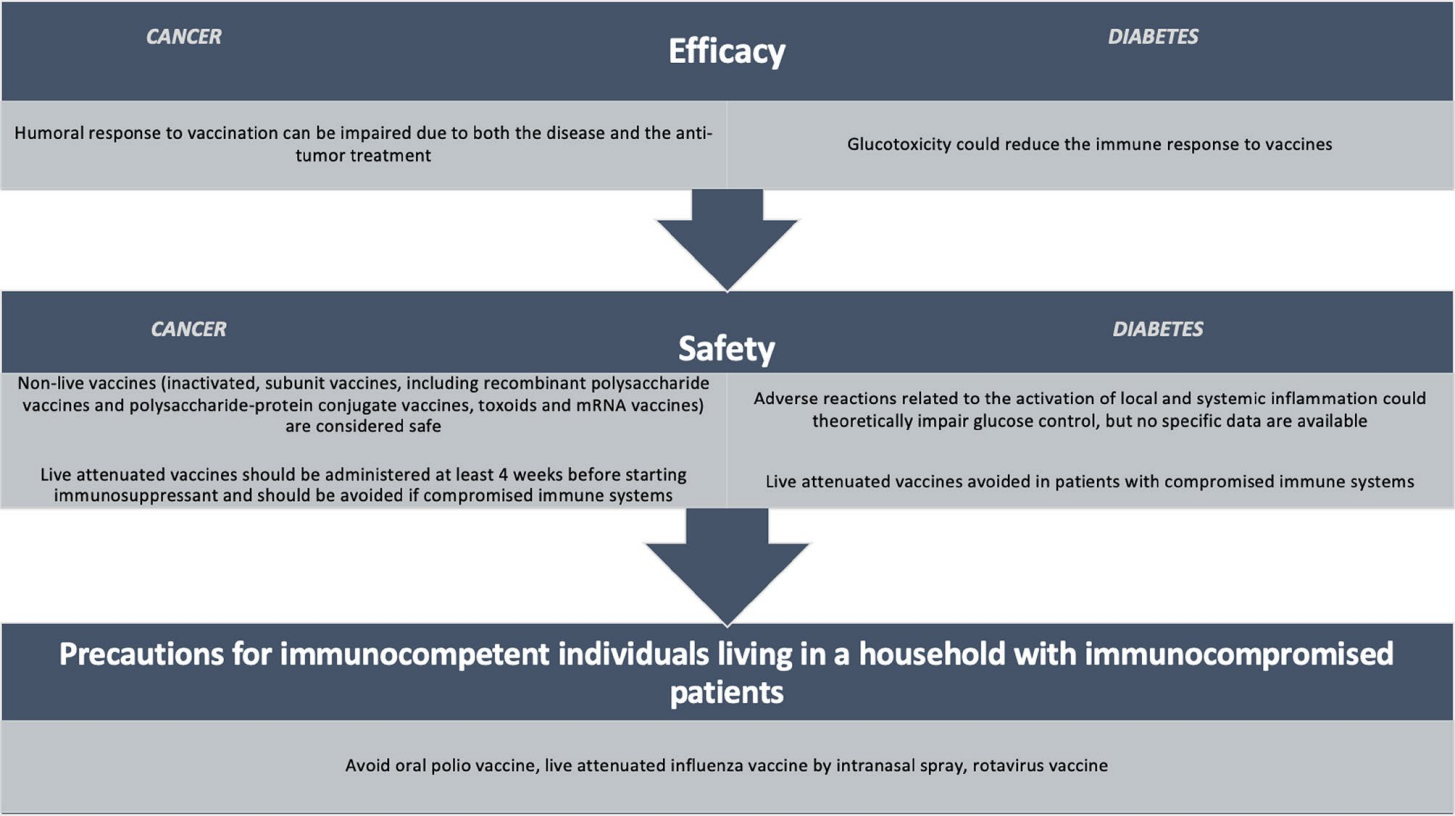
Efficacy, safety and precautions of vaccination in patients with cancer and diabetes

In cancer patients, non-live vaccines (inactivated vaccines, subunit vaccines, including recombinant polysaccharide vaccines and polysaccharide-protein conjugate vaccines, toxoids and mRNA vaccines) can be safely used. In contrast live vaccines, containing an attenuated but replication-capable virus or bacterium, may represent a risk of uncontrolled infection by the vaccine strain and are therefore to be avoided in cancer patients [4] (Fig. 2).

The effectiveness of vaccination is primarily assessed by the reduction in the incidence of infections. However, such studies do not usually provide detailed information on the reduction of infection rates due to the specific serum/genotype against which patients have been immunized [20]. This represents an important limitation. Furthermore, most data on vaccination in cancer patients derive from underpowered studies that include subjects with different tumor types and chemotherapy treatments and use different definitions of vaccine response. The efficacy of vaccination in cancer patients is related to the type of disease and the degree of immunosuppression [4], but also to the dosage and number of vaccine administrations [23].

Classically, vaccine response is measured by assessing antibody titers (immunoglobulin G) before and after vaccination [23]. In cancer patients, the humoral response to vaccination is often impaired due to both the disease and the anti-tumor treatment. However, other studies have recently found that the cellular response, and in particular the T-cell response, would be stronger than the humoral response, and possibly even more predictive of protection [24]. Regarding influenza vaccination, it has been reported that the response to the vaccine in cancer patients is similar to that of healthy patients [23]. Chiou WY et al. [25] demonstrated the efficacy of pneumococcal vaccination in elderly patients with colorectal cancer, with a significant reduction in the risk of hospital admission for pneumonia. The ASCO guidelines [4] on anti-COVID-19 vaccination state that seroconversion rates in adults with solid tumors tend to be lower than in those without tumors. Seroconversion rates are even lower in hematological diseases. Piubelli et al. [26] verified that COVID-19 vaccination is able to elicit a significant humoral response in patients with solid tumors, independent of tumor type and therapy. Furthermore, five non-randomized studies demonstrated that vaccination is useful in reducing severe COVID-19 disease in cancer patients (reduction in COVID-19 hospitalizations and death within 30 days compared to unvaccinated cancer patients) [27–31].

Regarding herpes zoster vaccination, the best humoral and cellular response after administration of the recombinant vaccine is expected immediately after tumor diagnosis and before the start of immunosuppressive therapy [4].

### Safety of vaccinations during cancer treatment

In general, inactivated vaccines are considered safe and should be administered at least 2 weeks before the start of chemotherapy and not during radiotherapy, to trigger an effective immune response. However, concomitant administration of inactivated vaccines with chemotherapy and radiotherapy is not harmful [23] (Fig. 2).

Live attenuated vaccines should be administered at least 4 weeks before the start of immunosuppressive therapy and should be avoided in patients with compromised immune systems [4]. The CDC and the IDSA recommend against vaccination with live attenuated vaccines for at least 3 months after stopping immunosuppressive therapy. Some chemotherapeutic agents cause immunosuppression beyond 3 months. For patients receiving regimens that include anti-B-cell antibodies, vaccination should be delayed for at least 6 months after the end of treatment. The ASCO guidelines [4] report that, in patients receiving immune checkpoint inhibitor (ICI) therapy, COVID-19 vaccination has similar seroconversion rates as in patients without cancer, with mild to moderate side effects (local pain and fatigue), and without any increase in immune-related adverse reactions in vaccinated patients. Influenza vaccination in patients undergoing ICI therapy also does not show an increased risk of immune-related adverse reactions compared to non-vaccinated patients undergoing therapy.

## Vaccinations in patients with diabetes

### Specific challenges in vaccinating patients with diabetes

Infections are associated with more severe complications in people with diabetes, compared to the general population, and may also increase blood glucose levels, thus making diabetes more difficult to manage. People with diabetes have a higher risk to be hospitalized for such complications [10]. In particular, a 6% increased risk of hospitalization was reported for seasonal influenza [32]. Conversely, patients who received influenza vaccination showed a lower risk of hospitalization for pneumonia [33]. In addition, in a retrospective study, influenza vaccination in patients with type 2 diabetes (T2D) was associated with a significant reduction in the risk of hospitalization for stroke, heart failure or pneumonia [34].

Therefore, children and adults with diabetes should receive all recommended vaccinations according to age-appropriate recommendations [12, 14, 35]. The European health authorities and the World Health Organization (WHO) set the target objective of 75% of vaccination coverage in the diabetic population [36]. The Italian Ministry of Health recommends and offers free influenza vaccination for people with diabetes, indicating a minimum vaccination coverage goal of at least 75% as desirable and 95% as the optimal goal. In the 2022–2023 flu vaccination campaign, vaccination coverage in the diabetic population was only 38%. The percentage improves when assessing the category of diabetic patients over 65 old years, reaching 72% coverage [37]. Although rates differ across nations, it has been reported that regular visits to primary care providers were associated with higher influenza vaccination rates among subjects with diabetes [38, 39]. Pneumococcal vaccine coverage data are not routinely collected. In elderly subjects coverage was relatively low, ranging from 0.7 to 50%, in different Italian regions [12]. Furthermore, despite the heavy burden of herpetic infection in immunocompromised people, current levels of anti- herpes zoster vaccine coverage in different countries are low in people with diabetes [40].

Elderly age, the presence of comorbidities, frequent primary care visits, and a previous history of vaccination have all been associated with greater vaccination adherence. Conversely, poor perception of the risk of infection complications, fear of adverse events, and a self-rated good health status have all been associated with lower vaccination adherence [41].

These considerations underline the specific challenges in vaccinating patients with diabetes.

Considering the gaps between recommendations and the real vaccination coverage, the following actions could be implemented to improve the compliance to vaccination in people with diabetes:

- analyzing the reasons why some people do not wish to receive vaccinations;
- removing barriers through education and providing information on the risks associated with infections and their effective prevention by vaccination;
- improving the dissemination work directed to both healthcare workers and people with diabetes, as well as their families.

A central role should be played by the collaboration of general practitioners with diabetologists in promoting access to vaccination. Various methods may be used to inform diabetic population, such as displaying posters in the waiting areas, providing adequate health education by healthcare professionals (physicians, nurses, and pharmacists), and sending vaccination reminders to both patients and their primary care providers.

Finally, national health institutions and scientific societies should collaborate to promote independent scientific research and information on vaccines, and on appropriate level of immunization in people with diabetes.

### Efficacy of vaccinations on immune response in patients with diabetes

Given the high risk for morbidity and mortality from infective diseases that people with diabetes experience, the WHO [42] and some international guidelines [14, 19] decided to strongly recommend several vaccinations in patients with diabetes.

One of the possible mechanisms underlying the greater susceptibility of patients with diabetes to infective diseases and their complications can be directly related to glucotoxicity [43]. Glucotoxicity could reduce the immune response to vaccines, as suggested by some human studies [44, 45]. An example of this putative lower efficacy of vaccination among patients with diabetes derives from hepatitis B vaccination, but several studies have shown reduced immunogenicity in patients with diabetes [46]. Nevertheless, some other authors reported that the humoral response of subjects with diabetes was not dissimilar from that of the general population [47, 48]. Moreover, some issues have been raised about vaccine safety in patients with diabetes [16]. Most adverse reactions are related to the activation of local and systemic inflammation, which could theoretically impair glucose control (Fig. 2). However, a recent observational study, performed on patients with type 1 diabetes (T1D) and assessing the effectiveness of mRNA-based anti-COVID-19 vaccine (Moderna), did not report any increase in glucose levels as shown by interstitial glucose monitoring data [49]. To further complicate the overall picture, the efficacy and safety of vaccines in people with diabetes could be theoretically different in distinct subpopulations (e.g., T1D vs. T2D, or different age groups); therefore, it would be very important to collect data on subgroups in order to issue evidence-based recommendations. Unfortunately, available studies do not report such subgroup analyses, limiting the information that can be collected in systematic reviews for clinical decision-making.

### Pneumococcal infections

Nowadays, two types of vaccines are available for protection against pneumococcal infections in adults: polysaccharide (23-valent: PPSV23) and conjugate (13-valent: PCV13; 15-valent: PCV15; 20-valent: PCV20) vaccines. All these vaccines fully demonstrated their efficacy in reducing pneumonia among immunocompetent adults [50, 51].

Little evidence is available on the effectiveness of anti-pneumococcal vaccination in patients with diabetes. A post hoc analysis of the only available randomized clinical trial [52] suggested that pneumococcal conjugate vaccine PCV13 is effective in reducing hospitalizations for pneumonia. On the other hand, a recent meta-analysis of observational studies failed to demonstrate the effectiveness of the pneumococcal vaccines in reducing hospitalizations and mortality in this population, irrespective of the type of vaccine used (PPSV23 and PCV13) [53]. Moreover, the same meta-analysis showed a likely reduction in the efficacy of the PPSV23 vaccine over time among patients with diabetes [53]. A previous systematic review exploring differences in pneumococcal-related outcomes in vaccinated adults with and without diabetes also provided conflicting results [54], and a previous meta-analysis including only patients with diabetes [55] raised some doubts about the efficacy of pneumococcal vaccines in this population. However, the only available randomized trial seems to suggest an effective protection of this vaccine also in patients with diabetes [52]. Moreover, it should be noted that all available evidence on the effectiveness of anti-pneumococcal vaccination derive from studies performed with PPSV23 and PCV13 vaccines. PCV15 and PCV20 are well-known to be more effective than previous vaccines [56]. Unfortunately, data on these vaccines among patients with diabetes are not yet available.

### Influenza

Data on the effectiveness of vaccination for influenza in patients with diabetes are more robust and convincing in comparison with those obtained for other vaccinations. There are several meta-analyses [33, 57] suggesting a similar efficacy in preventing adverse outcomes among individuals with diabetes. In particular, a recent systematic review and meta-analysis reported favorable results deriving from observational studies including a large cohort of patients with T2D, with long follow-up time, and with data on laboratory and clinical parameters [33]. This study observed an overall wide reduction in all-cause mortality associated with influenza vaccination, partly explained by the reduction in the risk of complications of influenza, such as pneumonia. In this analysis, influenza vaccination was associated with a reduced risk of hospitalization for pneumonia. However, other mechanisms cannot be ruled out: for example, among patients with cardiovascular disease, influenza vaccination can reduce cardiovascular mortality and morbidity [58]. Moreover, infections such as influenza can increase blood glucose levels, acutely worsening diabetes and its intercurrent complications [59].

### Herpes Zoster

Patients with T2D show an increased risk of both herpes zoster (HZ) infection and complications, and a further increase in risk has been observed in older people, especially with associated cardiovascular disease [60, 61]. A recent systematic review and meta-analysis of the few available clinical trials and observational studies assessed the efficacy and effectiveness of HZ vaccines in people with diabetes. According to this analysis, the quality of evidence for efficacy of the recombinant zoster vaccine (RZV) was higher than that for the live attenuated vaccine, even if no head-to-head comparison between the two available vaccines has been performed in people with diabetes [62].

## Patients with cancer and diabetes

### Immunological vulnerabilities in patients with cancer and diabetes

Diabetes and cancer are among the leading causes of death worldwide, and their incidence and prevalence have progressively increased in recent years [63]. A strong interconnection between these two medical conditions has been described, with patients with diabetes being at higher risk to develop some malignancies [64], on the one hand, and anti-cancer treatments potentially leading to iatrogenic hyperglycemia and/or diabetes-related complications [15, 65], on the other. A negative impact of diabetes on cancer-related mortality has also been reported [65, 66]. A common denominator between diabetes and cancer is represented by immune system disfunction, which exposes affected patients to higher risk of infections.

Indeed, cancer patients often have a compromised immune system due to various factors, such as chronic inflammation and toxicities from anti-cancer treatments (e.g. chemotherapy and radiotherapy) [4]. Diabetes, as well, is associated with impaired T-cell responses, decreased neutrophil function, and reduced humoral immunity [67]. Several studies have demonstrated that patients with T2D and hyperglycemia show increased rates of bacterial infections, fungal infections such as candidiasis and mucormycosis, as well as shorter remission periods, shorter median survival times, higher rates of hospital admissions, more infection-related admissions, and higher mortality rates [68, 69]. A systematic review and meta-analysis revealed that preexisting diabetes in cancer patients was associated with an increased risk of all-cause mortality compared to cancer patients without diabetes [70].

The immune system function is a major factor in determining the spectrum of infections to which cancer patients are more vulnerable. Host defense mechanisms are mediated by the immune system, which is traditionally divided into the innate (general or non-specific) compartment and the adaptive (specialized or specific) one [71–73]. The former is constitutively present, not antigen-specific, and able to mobilize rapidly, thus providing the first line of defense for invading microorganisms. The innate immune system is comprised of anatomical barriers, humoral factors that aid in the inflammatory response, and cellular components that facilitate phagocytosis. In cancer patients, these barriers can be compromised by malignant invasion, mechanical obstruction, or treatments such as radiation and cytotoxic chemotherapy. The adaptive immune system is antigen-specific and exhibits immunological memory. Therefore, it requires time to react but can react more rapidly, although not as quickly as innate immunity, on repeated exposure to the same organism [72]. Adaptive immunity includes both humoral and cellular components mediated by B and T lymphocytes, respectively.

Both the innate (general) and the adaptive (specialized) immune systems may be altered in cancer patients. Factors that predispose to infection are divided into “host-associated” and “treatment-associated”. Host-associated factors include underlying immune deficiencies, medical comorbidities, past infections, poor nutritional status, presence of foreign bodies, and psychological stress. Treatment-associated factors include surgery, radiation, immunosuppressant therapies, antimicrobial use, and invasive procedures [71].

For instance, chemotherapy and radiation therapy may result in decreased number of circulating neutrophils. A subset of solid tumor patients receives intensive chemotherapy that is complicated by neutropenia. Moreover, anticancer therapy further increases the risk of infection by causing delayed wound healing and mucosal lesions [74].

Finally, recent evidence suggests that the patient’s microbiota could also affect different aspects of carcinogenesis, favoring the proliferation of epithelial cells, establishing inflammatory microenvironment, and promoting treatment resistance [75].

### Impact on oncological outcomes

In the last few decades studies on the relationship between diabetes and cancer have increased exponentially [76–80]. A meta-analysis of 151 cohorts indicated a strong causal association between T2D and the incidence of liver, pancreatic, and endometrial cancer, and a reduced risk of prostate cancer [81]. Less clear is the relationship between T1D and the incidence of cancers, with more controversial data [82]. Cancer patients with diabetes have a worse prognosis [65, 83], probably also because high glucose levels induce an intracellular signaling alteration in tumor cells with a consequent more aggressive behavior [84]. Several studies in different types of cancer demonstrated that hyperglycemia can upregulate the VEGF/VEGFR pathway and promote neoangiogenesis [85]. Moreover, high glucose promotes the upregulation of epithelial-mesenchymal transition (EMT) transcription factors in different modalities depending on the specific type of cancer [86, 87]. Finally, hyperglycemia influences the chemoresistance in various cancers via multiple modalities, such as the upregulation of insulin-like growth factor binding protein-2 (IGFBP-2) in breast cancer [88] and the SMAD3 and MYC phosphorylation in colorectal cancer [89]. The impact of diabetes on cancer therapies also seems to apply to immunotherapy. A recent study has revealed that ICIs seem less beneficial in patients with non-small cell lung cancer (NSCLC) and diabetes with a significantly shorter median progression-free survival (PFS) and overall survival (OS), as compared to patients without diabetes [90].

### Impact on diabetes outcomes

In recent years, advances in diabetes management and the resulting increase in life expectancy of patients with diabetes have allowed to identify new comorbidities, defined as emerging complications of diabetes, including cancer [91]. Furthermore, the increase in the incidence of diabetes is paralleled by the increasing incidence of cancer [92] and it is estimated that approximately 20% of people with cancer have or will develop diabetes, more than double the incidence of diabetes in the global adult population [93, 94]. As a consequence, in some countries, cancer has become the leading cause of mortality in people with diabetes [95, 96].

The presence of cancer in patients with diabetes also has a significant impact on diabetic outcomes. For instance, tumor cachexia, defined as a multifactorial syndrome characterized by loss of skeletal muscle (a crucial organ in the maintenance of glucose homeostasis and among the main targets of insulin action), is often associated with glucose intolerance, insulin resistance, and inflammation, which predispose to T2D development or worsening [97, 98]. In addition, cancer-related stress (especially due to acute illnesses, recurrent hospitalizations, surgeries, infections, and hemorrhages) can also induce or worse hyperglycemia and inflammation [97]. Finally, when cancer affects organs involved in glycemic homeostasis, such as pancreas and the liver, it can cause diabetes as a direct consequence of the altered functioning of these organs or secondary to the resection of the tumor (e.g., after partial or total pancreatectomy) [92]. In patients with diabetes and cancer, management of diabetes should be tailored to the patient, individualizing glycemic targets, glucose monitoring, and treatment goals, usually prioritizing the continuation of cancer treatment [99–101]. Indeed, cancer treatment is associated with decreased diabetes medication adherence and self-management behaviors, such as blood glucose monitoring [102, 103]. In addition, several anti-cancer therapies, such as corticosteroids [104], somatostatin analogs (SSAs) [92], mTOR inhibitors [15, 101], and ICIs can directly affect glucose homeostasis, thus increasing the risk of new-onset hyperglycemia or worsening of pre-existing diabetes, posing significant difficulties for its management [105, 106]. The mechanisms underlying these effects are variable. Corticosteroids can cause hyperglycemia and diabetes by increasing hepatic gluconeogenesis and inhibiting glucose uptake into adipose tissue [104]. SSAs, acting on somatostatin receptors, induce hyperglycemia and diabetes through a direct suppression of insulin secretion by pancreatic beta-cells and of GLP-1 by enteroendocrine L cells [92, 107, 108]. Although it is not entirely known how ICIs can determine the onset of diabetes, it has been proposed that these drugs evoke an immune response against pancreatic beta-cells by activating the immune system [109].

### Synergistic effects of cancer and diabetes on the immune response

**Fig. 3:**
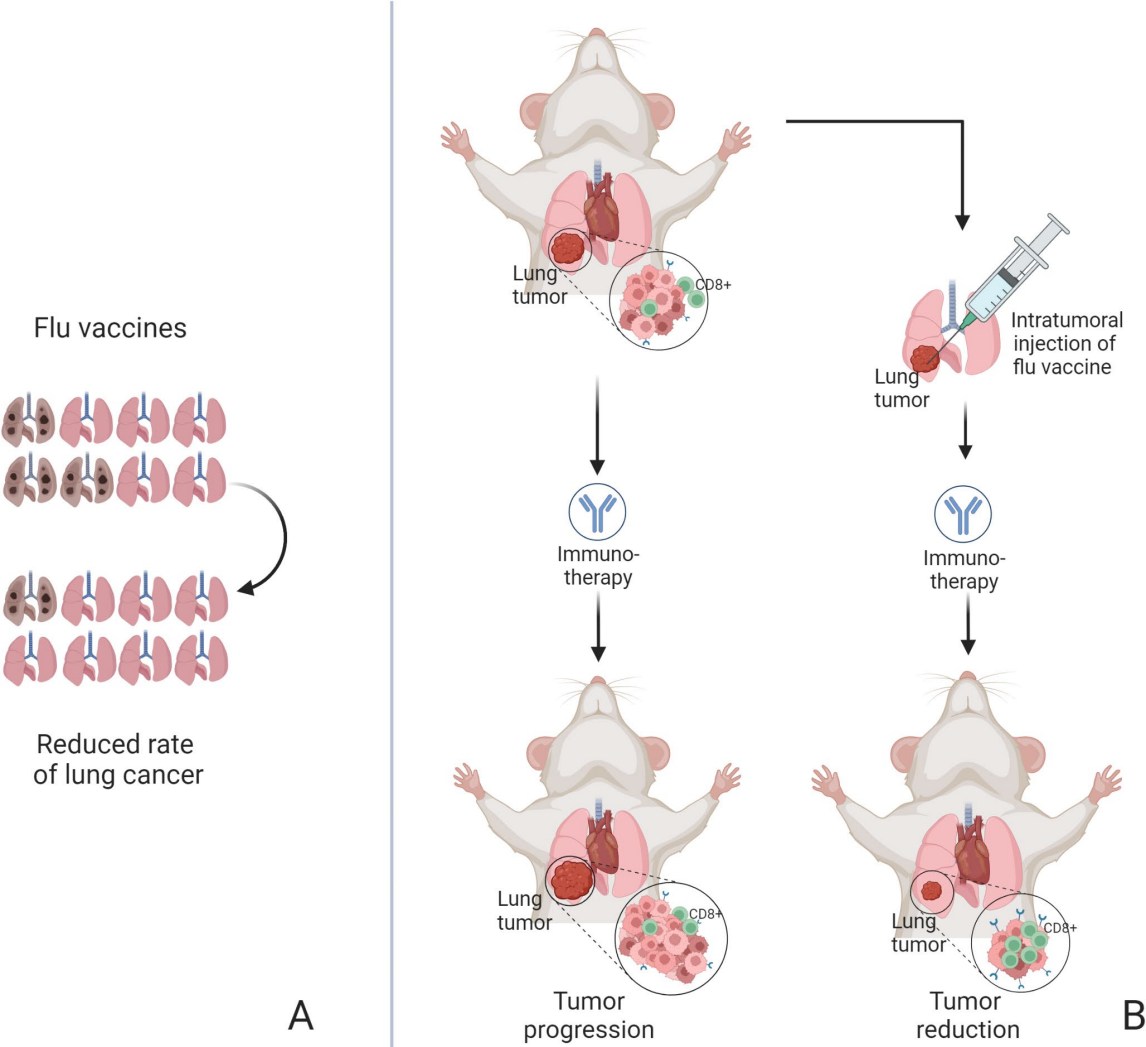
(**A**) Flu vaccine significantly reduces the risk of lung cancer in patients with diabetes. (**B**) Administration of flu vaccine by intra-tumor injection in mice with lung cancer sensitizes tumors to PD-L1 checkpoint inhibitors. Created with BioRender.com

To the best of our knowledge, there are no studies investigating the potentially negative and synergistic effect of diabetes and cancer on the immune response, and on vaccination effects. Efficacy and safety of vaccines are often analyzed independently in the two conditions. Indeed, a large cohort study showed that the humoral immune response to COVID-19 mRNA vaccines was good both in patients with cancer and in those with diabetes. Compared to the control group, however, the strength of the humoral neutralizing response in these two categories was reduced [110]. Another cohort study investigated the immunogenicity of the ChAdOx1 nCoV-19 vaccines in subjects with different comorbidities, finding cancer -but not diabetes- to significantly impair immune response [111]. However, a group of patients with both diabetes and cancer was not included in these studies, and cellular mechanisms were not investigated. Nevertheless, an interconnection between diabetes, cancer, and immune response has been hypothesized. A cohort study showed that annual flu vaccine significantly reduced the risk of lung cancer in patients with diabetes, with a dose-dependent protective effect [112] (Fig. 3A). This evidence was further supported by a preclinical study, where administration of flu vaccine by intra-tumor injection in mice with lung cancer increased CD8 + T cells within the tumor microenvironment, reduced tumor growth, and sensitized these tumors to PD-L1 checkpoint blockade treatment [113] (Fig. 3B). In keeping with this evidence, the CD8 + T lymphocytes role seems crucial. Indeed, circulating CD8 + PD-1 T lymphocytes display lower basal mitochondria respiration, reduced glycolysis, and lower count in T2D patients. These latter findings could be implicated in greater tumor progression and susceptibility to viral infections in T2D patients, as compared to the general population [114]. In addition, tumor-infiltrating CD8 + T cells in patients with diabetes and colorectal cancer display a different transcriptomic profile, as compared to those of non-diabetic patients with colorectal cancer, involving genes in common between diabetes, cancer, and immune response such as LGALS1, CDKN2 A, B3GALT4 [115].

Based on the reported evidence, we can hypothesize that in patients with both diabetes and cancer the immune response to vaccines could be reduced, as compared to patients without these two conditions. However, studies considering patients with both conditions undergoing vaccinations are lacking and strongly warranted, since these patients are frequent in daily clinical practice. Vaccine pleiotropic effects should be investigated to evaluate their efficacy, safety, and adverse events in this setting, since they could modify patient’s outcome.

## Recommendations and guidelines

### Recommended immunization strategies

A strong mutual relationship exists among most of vaccine preventable diseases (VPDs) and disease course of both diabetes and cancer [3]. In recent years, medical oncology and diabetology international scientific societies published several guidelines to define and promote vaccination strategies in patients, respectively, with cancer or diabetes. Nonetheless, the patient’s adherence to these recommendations, as well as clinician’s perception of the clinical relevance of this topic, are still scant [116].

Many vaccination schedules are substantially similar in both people with cancer and with diabetes. Disease, age, and schedule of administration of vaccines that should be offered to patients with both diabetes and cancer are summarized in Table 1, as finally agreed by the working group from a multidisciplinary panel of Italian scientific societies.

**Table 1:** Recommended immunizations for adults affected by Diabetes and cancer

Vaccines	Recommended ages	Schedule	
Influenza	All ages	Annual, with (preferably high dose) inactivated quadrivalent vaccine. Live-attenuated vaccines are contraindicated	
COVID-19	All ages	Initial vaccination and booster, according with National Health Care System schedule*	
Pneumonia	19 years and older	Initial priming with a conjugate vaccine (15-valent pneumococcal conjugate vaccine, PCV15, or 20-valent pneumococcal conjugate vaccine, PCV20) followed at least 8 weeks later by a dose of 23-valent pneumococcal polysaccharide vaccine (PPSV23)	
Zoster	19 years and older	Two doses at least 4 weeks apart	
RSV	60 years and older	Single dose	
Hepatitis B**	All people 19–59 years old	Three-dose Recombivax HB series (0, 1, 6 months) can be proposed	
	60 years and older: immunize those with other risk factors	Check antibody surface titer 1–2 month after the last dose; if < 10 mUI/ml, repeat vaccination schedule	
Tetanus, diphtheria, pertussis (Tdap)	All adults	One dose of Tdap, followed by Td or Tdap booster every 10 years	
HPV	19–26 years: eligible	Two or three doses schedule	
	27–45 years: *shared decision making*		

The table summarizes disease, age, and schedule of administration of vaccines that should be offered to patients with Diabetes and cancer. When differences have been found among available guidelines, the working group discussed the evidence and provide a final recommendation, according to a prudent principle and giving greater consideration to the condition (i.e. Diabetes or cancer) with higher potential clinical impact in the specific VPD

*Evaluate immunosuppression grade; **if negative screening for HBV

Both cancer and diabetes can be considered as chronic disease, inducing frailty and disability in affected subjects and with increasing impact on health care systems [117]. A clear and tailored communication can be of great value to resolve patients and caregivers doubts and increase the acceptance of vaccines. This effort could represent a valid tool to help health care systems to mitigate the negative impact of VPDs on patients with cancer and diabetes.

### Oncological guidelines

AIOM was one of the first scientific societies to publish recommendations on vaccination against VPDs in cancer patients, specifically flu, in 2014 [5]. Since then, AIOM has continued to promote vaccinations, increasing oncologists’ awareness of anti-pneumococcal [3, 118], anti-SARS-CoV-2 [3], and anti-Herpes Zoster [119] vaccines. Moreover, AIOM has recently investigated oncologists’ knowledge of this issue with a survey with disappointing results: only 30% of respondents discuss vaccinations with patients during the first oncological visit and only 44% of them consider vaccination an issue of their pertinence in clinical practice [116].

The main European and international scientific societies agree that vaccination counseling is important at the time of patient referral with vaccine schedule planning, ideally before the initiation of cancer treatments [3, 4]. Scientific societies recognize that the diagnosis of cancer is a devastating event and that vaccination is not perceived as an immediate priority by the patient (and often not even by the oncologist). However, several studies demonstrated that, when vaccines are administered before starting oncological treatment, the best protection is achieved. To achieve this outcome, vaccination counseling should represent an integral part of the management of cancer patients, through multidisciplinary team and allocated resources.

Vaccination history should be mandatorily listed on the medical record, in order to easily identify which vaccinations are missing or which need to be updated with seasonal booster shots (e.g. anti-flu and anti-SARS-CoV-2).

Active collaboration with general practitioners, hygienists, and pharmacists is necessary to implement the vaccination offer, possibly by organizing dedicated outpatient facilities within the same hospital, if feasible.

Vaccination counseling should also be extended to family contacts (caregivers) to better protect patients (cocooning vaccination).

Live-attenuated vaccines should be avoided in cancer patients because they may induce uncontrolled infection from the vaccine strain. For this reason, measles, mumps, and rubella (MMR) vaccine, oral typhoid, and Monkeypox and smallpox (ACAM2000) vaccines are contraindicated during cancer treatment. Non-live vaccines are safe for use in cancer patient and can be administered before and/or during oncological treatments. Examples of non-live vaccines are inactivated vaccines, recombinant and polysaccharide-protein conjugate subunit vaccines, and mRNA vaccines.

### Influenza vaccines

Most infection-related deaths are attributed to influenza and pneumonia deaths that can be prevented by vaccination. The mortality rate of fatal infections in cancer patients is nearly three times that of the general population [SMR, 2.92; 95% (confidence interval) CI 2.91–2.94] [120]. Studies support the safety and benefits of influenza vaccination in reducing the severity of infections and associated hospitalizations, while recognizing a lower rate of seroconversion compared to immunocompetent individuals [121, 122]. Cancer patients should annually receive one of the preferentially recommended high-dose or adjuvanted vaccine formulations (high-dose Quadrivalent vaccine, and Quadrivalent adjuvanted flu vaccine) according to their availability [123, 124].

### COVID-19 vaccines

Cancer patients show a higher rate of complications and mortality during COVID-19 compared to healthy subjects, with poorer outcomes in hematologic malignancies and lung cancer [125, 126].

In cancer patients, there is a suboptimal seroconversion rate after COVID-19 vaccination when compared with immunocompetent subjects [127, 128]. According to the diminished immunogenicity and the continuous evolution of the virus into new variants, several studies state that annual boosting may assure the optimal protection in immunocompromised patients [129, 130]. The vaccination can reduce the risk of severe COVID-19 illness [27], the rates of hospitalization and mortality [28], and the risk of COVID-19 sequelae (long COVID) [29]. The VAX4 FRAIL study reported clinically manageable adverse events toxicities [131]. Transient axillary adenopathy can appear after COVID-19 vaccination, for this reason the Society of Breast Imaging has published recommendations on this topic [132].

### Pneumococcal vaccines

*Streptococcus pneumoniae* (*S. pneumoniae*, pneumococcus) can cause severe pneumonia and meningitis in immunocompromised subjects [133]. The spread of pneumococcal resistant clones to the common antibiotics represents a growing issue [134]. The immune response to the pneumococcal vaccine on the day of chemotherapy or 2 weeks before has been demonstrated similar in a small prospective randomized controlled trial in patients with gastrointestinal cancer undergoing chemotherapy [135]. The pneumococcal vaccine is useful to reduce the risk of pneumonia-related hospitalization in cancer patients aged ≥ 75 years [136].

The currently recommended approach is an initial priming with a conjugate vaccine (15-valent pneumococcal conjugate vaccine, PCV15), followed at least 8 weeks later by a dose of 23-valent pneumococcal polysaccharide vaccine (PPSV23) [12] or a single dose of 20-valent pneumococcal conjugate vaccine (PCV20) only [12].

### Recombinant Zoster vaccines

The incidence of HZ is high in the first 2 years after cancer diagnosis, especially in patients younger than 50 years of age. Complications of HZ, such as post-herpetic neuralgia (PHN), can negatively impact quality of life [137].

The approval of the adjuvanted, RZV by the US Food and Drug Administration (FDA) in 2017 converted HZ into a VPD [138]. The vaccine schedule consists of two doses at least 4 weeks apart. Humoral responses are higher when RZV is administered before chemotherapy, rather than on therapy [139]. An increase of T cell response was observed in 67.5% cancer patients undergoing immunotherapy both 28 days after the second dose and six months after the second dose [140], confirming the immunogenicity of the vaccine even during ICIs treatment.

### Respiratory syncytial virus (RSV) vaccines

RSV clinical manifestation ranges widely from mild upper respiratory infections to severe lower respiratory tract infections (LRTIs) [141]. There are no specific studies on this vaccine in patients with cancer. Therefore, awaiting specific data on this population, the recommendation for the general population remains valid. Patients with lung and mediastinal cancer and patients with lung metastases are at increased risk, so it is recommended to prioritize this population.

### Hepatitis B vaccines

All cancer patients should be tested for Hepatitis B Virus (HBV) before starting any systemic oncological therapy by 3 tests (hepatitis B surface antigen [HBsAg], hepatitis B core antibody [anti-HBc], and antibody to hepatitis B surface antigen). HBV reactivation risk assessment is based on the diagnosis of chronic HBV (HBsAg-positive) or past HBV (HBsAg-negative and anti-HBc-positive) infection [142]. If serologic assessment for HBV infection is negative, a three-dose Recombivax HB series (0, 1, 6 months) can be proposed to the patient [143]. Post-vaccination antisurface antibody titers should be checked 1–2 months after the last dose, and if hepatitis B surface antibody concentrations are lower than 10 mUI/mL, the entire vaccine schedule should be repeated.

### Monkeypox

Mpox (formerly monkeypox) is a viral disease caused by monkeypox virus (MPXV), genus Orthopoxvirus. The clinical manifestation of the disease includes general symptoms such as fever and headache, and a frequent and characteristic rash (papules, vesicles, and pustules), often with concomitant mucous (oral cavity) lesions. Severe pictures of the disease may manifest as visceral localization and bacterial superinfection. Immunocompromised people, especially untreated HIV-infected persons with low CD4 counts, are at risk of developing complications and death due to mpox. The only mpox vaccine authorized by the EMA is the modified live Ankara vaccine virus - Bavarian Nordic (MVA-BN). It is safe to administer in immunocompromised patients, such as people with cancer and/or HIV.

Mass vaccination for mpox is currently neither required nor recommended. At present, vaccination is offered to some at-risk groups and travelers to endemic areas [144]. 

### Diphtheria, tetanus, pertussis (Tdap)

Diphtheria-tetanus-pertussis vaccination (Tdap) is recommended for people aged 7 and over. Adults should receive a booster dose of Tdap every 10 years, or after 5 years in case of severe or dirty wounds [145].

### Human papillomavirus (HPV)

Persistent infection with HPV strains with a high carcinogenic risk, in particular HPV 16 and HPV 18, is responsible for the occurrence of carcinomas of the anogenital and oropharyngeal region. Infections with low-risk carcinogenic strains, such as HPV 11, are responsible for more than 90% of condylomas in the anogenital area. Most HPV-associated cancers are preventable by vaccination [146]. The target populations for HPV vaccination are all subjects of both sexes aged between 9 and 12 and 26 years. The indication may be extended up to the age of 45 for subjects with low HPV exposure, with a previous incomplete vaccination cycle, sex workers, men who have sex with men, and transgender and gender-diverse. The HPV vaccine is most effective if administered before the start of sexual activity [147].

### Diabetological guidelines

People with diabetes are at higher risk for developing infections compared to the general population, and the course of the infection is more complicated [148, 149]. The recent COVID-19 pandemic has corroborated the close association between infections and diabetes, following observations that people with diabetes are more likely to progress to severe COVID-19 disease and die than those with normal glucose metabolism [150]. Some common infections are preventable through vaccination and international guidelines recommend routine vaccination for adults with diabetes [14].

### Influenza vaccines

Influenza is a common and preventable infectious disease associated with high mortality and morbidity, particularly among the elderly and individuals with chronic conditions. Influenza vaccine reduces all-cause mortality by 50% and lowers the risk of hospitalization for pneumonia by 11% [33, 151]. Consequently, the ADA recommends annual influenza vaccination for all individuals with diabetes [14].

Influenza vaccination is recommended for all individuals ≥ 6 months of age without contraindication, preferably using an inactivated quadrivalent vaccine (QIV). The live attenuated influenza vaccine, which is delivered by nasal spray, is an option for people who are aged 2–49 years, but people with diabetes are cautioned against taking the live attenuated influenza vaccine and are instead recommended to receive the inactive or recombinant influenza vaccination. For individuals > 65 years, the high-dose quadrivalent inactivated influenza vaccine may offer additional benefits [124].

### COVID-19 vaccines

People with diabetes are more likely to develop severe symptoms and complications in case of SARS-CoV-2 infection [152]. Hospitalization and mortality rates are higher than the general population and further worsened in the event of poor glucose control [153]. Therefore, subjects with diabetes should receive all recommended doses of SARS-CoV-2 vaccine including the required boosters [14, 35].

### Pneumococcal vaccines

People with diabetes show higher rates of pneumococcal infection as well as a higher risk of pneumonia-related hospitalization and mortality [154]. Both the pneumococcal conjugate vaccine (PCV) and the polysaccharide vaccine (PPV) have been shown to be effective in subjects with diabetes [52, 155, 156]. Both the ADA and the Italian diabetes societies [35] recommend that adults aged 65 or older, as well as adults aged 19–64 with underlying risk factors or severe comorbidities, whose vaccine status is unknown or who have not received pneumococcal vaccine, should receive one dose of PCV15 o PCV20. According to the recommendations of the CDC, If PCV15 is used, it should be followed by PPSV23 after at least one year [14, 35]. Adults previously immunized with PCV13 should receive one dose of PPVS23 after one year, too [157, 158].

### Recombinant Zoster vaccines

People with diabetes are at a higher risk of HZ infection, particularly women, elderly individuals, and those with T1D [40]. Two HZ vaccines are currently available: the RZV and the zoster vaccine live (ZVL). Both vaccines are effective in preventing HZ, with ZVL additionally reducing the risk of postherpetic neuralgia [159]. RZV [160] is recommended for individuals over 50 years of age, with two doses administered within two months [14]. Additionally, it is advisable to consider revaccination with RZV for individuals who have already been vaccinated with the live attenuated vaccine, after a minimum interval of eight weeks [160].

### RSV vaccines

RSV infection is an important illness in elderly and high-risk adults. Patients with diabetes aged 60 or older are considered as a high-risk group [161]. According to the recommendations of the ACIP Respiratory Syncytial Virus Vaccines Adult Work Group and to the ADA, all adults aged 60 or older should receive a single dose of an RSV vaccine [162].

### DPT vaccine

There are no specific indications regarding this vaccination for individuals with diabetes. DPT vaccination is recommended for adults aged 19 to 64 years, with periodic administration every 10 years. Additionally, the vaccine is recommended during pregnancy, and the booster dose should be repeated with each pregnancy, even if pregnancies are closely spaced. The presence of diabetes in pregnant women is not a contraindication for vaccination [163].

### Hepatitis B vaccine

Compared with the general population, people with diabetes have higher rates of hepatitis B infection [14]. According to the ADA, the hepatitis B vaccine is recommended for adults with diabetes aged < 60 years. For adults aged ≥ 60 years, hepatitis B vaccine may be administered at the discretion of the treating clinician.

The Italian SID-AMD standards recommend vaccination for all unvaccinated individuals with diabetes. Additionally, periodic screening of the anti-HBs antibody titer is advised, along with the administration of booster doses for those with a decline in antibody levels [164]. Some studies indicate that patients with diabetes may achieve a lower protective antibody response or produce a quantitatively lower antibody response to the hepatitis B vaccine compared to healthy individuals [165, 166].

### Papilloma virus vaccine

There are no specific indications regarding this vaccination for individuals with diabetes. HPV vaccination is recommended for individuals < 26 years, with three doses for males and two doses for females over a six-month period. Individuals > 26 years may receive the HPV vaccine after consulting with healthcare professionals [167].

## Strategies for overcoming challenges to vaccination in patients with cancer and diabetes

Patients with cancer and diabetes face unique challenges regarding vaccination due to their compromised immune systems, and overcoming these challenges requires a multifaceted approach. Raising concerns for vaccine hesitancy are multifactorial, including the optimal timing of vaccination, doubts about efficacy, and potential adverse effects of vaccines in immunocompromised patients. It has been widely recognized that both cancer treatments and diabetes can weaken the immune system, leading patients and/or caregivers to worry about vaccine efficacy and safety. Accordingly, it is widely recognized that patients with B cell malignancies (particularly myeloma) and those receiving anti-CD20 monoclonal antibodies have the weakest humoral responses. Although international guidelines recommend inactivated influenza vaccination in this specific population based on data supporting efficacy and excellent safety profiles, outcome has often been suboptimal due to persisting hesitancy among both patients and oncologists regarding the optimal vaccine schedule and timing, and the best method to assess response in immunocompromised populations.

**Fig. 4:**
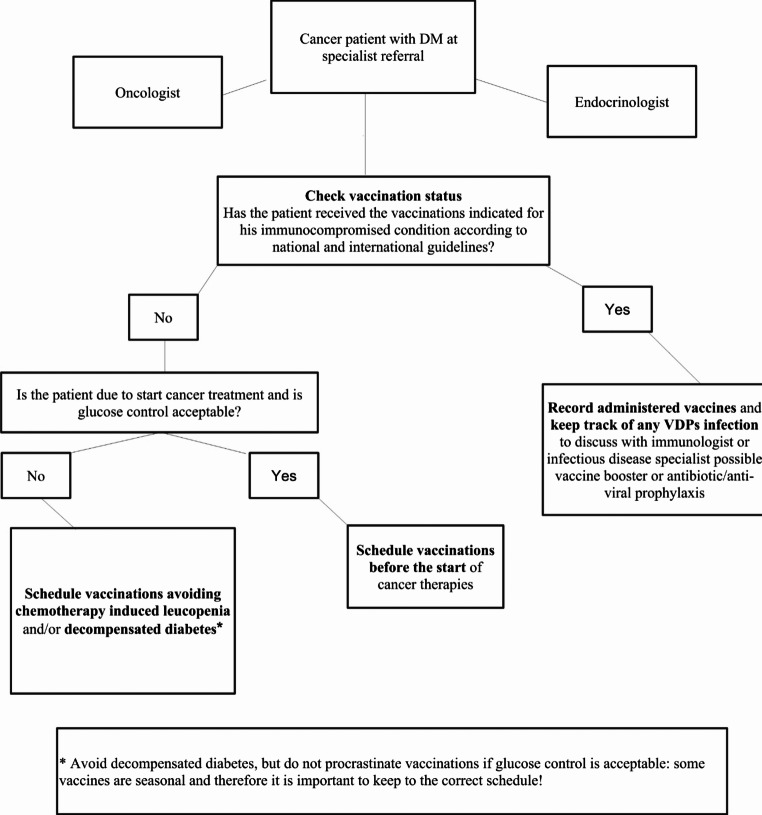
Tentative decision-making algorithm for vaccinating cancer patients with diabetes

Coordination between diabetologists and oncologists is essential for determining the best timing for vaccination. Indeed, scheduling vaccinations during periods of lower immunosuppression and routine diabetes check-ups can increase patients’ compliance while optimizing vaccine efficacy. Furthermore, well-controlled blood glucose levels before vaccination can also improve outcomes [168] (Fig. 4). Cancer treatments often involve a complex regimen of medications, including drugs used to mitigate side effects and/or to treat comorbidities other than diabetes. This can increase hesitation since some patients believe that vaccines might affect their treatment outcomes. Strategies to improve vaccination rates may also rely on tailored communication with and education of patients. Healthcare providers should have empathetic conversations with patients to address their specific concerns, use simple, non-technical language to explain the benefits and safety of vaccines, and provide easily understandable evidence-based written materials and visual aids. Training healthcare providers on vaccination guidelines for immunocompromised patients may also ensure they will be well-equipped to address patient concerns and questions.

Using inactivated vaccines instead of live vaccines and choosing vaccines with a proven safety record in immunocompromised populations will significantly reduce the risk of infection, while ensuring safe and effective vaccination practices. These vaccines should be part of immunization programs for patients undergoing cancer treatment and those with chronic conditions like diabetes. Health organizations (e.g., CDC and WHO) and scientific societies (e.g., ADA, ASCO, and IDSA) provide guidelines for vaccinating immunocompromised individuals that emphasize the preference for inactivated vaccines, offering recommendations on timing and dosing to optimize safety and efficacy.

Other important issues are closely monitoring patients after vaccination for adverse reactions and communication with patients about recognizing potential adverse events, and how to manage them. Improving access to vaccination using mobile units and home vaccination programs is pivotal to reaching patients with limited access to healthcare facilities. Finally, supportive policies that ensure insurance coverage for recommended vaccines and funding of vaccination programs targeting high-risk populations may remove financial barriers and improve access to needed vaccines.

With the increase in life expectancy of cancer patients, it is important for oncologists to preserve the best quality of life of their patients. Effective prevention of the main VPDs through vaccinations is important from an oncological, social-health, and economic standpoint. Oncologists have a key role in educating their patients on the importance of vaccinations to prevent VPDs and ensuring the effectiveness and safety of oncological treatments. Targeted education campaigns and multidisciplinary collaboration between oncologists and diabetologists would be paramount to filling gaps in current evidence and areas while improving vaccination uptake.

## Conclusions

Diabetes and cancer are becoming more and more common worldwide and, given their involvement in multiple aspects and functions of the body, their management implies a “holistic” approach by different professional figures. Prevention of communicable infectious diseases represents one of these aspects, considering the deep impact of both diabetes and cancer (and their therapies) on the immune system and, consequently, on the infection risk. Vaccination proved safe and effective in reducing the risk of infection and adverse consequences of infectious diseases, both in people with diabetes and in patients with cancer. Taking into account the synergistic negative effect on the immune system of both conditions, vaccination in cancer patients with diabetes represent an even more important aspect of clinical management.

This position statement summarizes both oncological and diabetological guidelines in order to provide clinicians (oncologists, diabetologists, and every professional figure involved in the management of these patients) a concise and helpful outline of the main vaccinations recommended for patients with cancer and diabetes. Many aspects of this matter are, however, still controversial, like the optimal administration schedule (particularly in relation to chemo/radiotherapy schedules or other oncological therapies that could further derange the immune system function), the strategies for overcoming potential suboptimal response in immunocompromised patients, and the logistic challenges faced by health institutions for organizing vaccination sessions for a vast number of patients and their caregivers. New research findings will be important for overcoming these challenges and controversies, with the aim of further optimizing the management of cancer patients with diabetes and improving their outcomes.

## Electronic supplementary material

Below is the link to the electronic supplementary material.

Supplementary Material 1

## Data Availability

Not applicable.

## References

[1] World Health Organization (2024) Development of WHO immunization policy and strategic guidance: methods and processes applied by the Strategic Advisory Group of Experts on Immunization (SAGE) to develop evidence-based recommendations

[2] Doherty M, Schmidt-Ott R, Santos JI et al (2016) Vaccination of special populations: protecting the vulnerable. Vaccine 34:6681–6690. 10.1016/j.vaccine.2016.11.015 27876197 10.1016/j.vaccine.2016.11.015

[3] Pedrazzoli P, Lasagna A, Cassaniti I et al (2023) Vaccination for seasonal influenza, Pneumococcal infection and SARS-CoV-2 in patients with solid tumors: recommendations of the associazione Italiana Di oncologia medica (AIOM). ESMO Open 8:101215. 10.1016/j.esmoop.2023.101215 37104930 10.1016/j.esmoop.2023.101215 PMC10067463

[4] Kamboj M, Bohlke K, Baptiste DM et al (2024) Vaccination of adults with cancer: ASCO guideline. J Clin Oncol 42:1699–1721. 10.1200/JCO.24.00032 38498792 10.1200/JCO.24.00032 PMC11095883

[5] Pedrazzoli P, Baldanti F, Donatelli I et al (2014) Vaccination for seasonal influenza in patients with cancer: recommendations of the Italian society of medical oncology (AIOM). Ann Oncol 25:1243–1247. 10.1093/annonc/mdu114 24618150 10.1093/annonc/mdu114 PMC7109906

[6] ESMO Supporting policies to boost vaccination coverage against cancer-causing viruses. https://www.esmo.org/policy/vaccine-preventable-cancers. Accessed 8 Sep 2024

[7] Abu-Ashour W, Twells L, Valcour J et al (2017) The association between diabetes mellitus and incident infections: a systematic review and meta-analysis of observational studies. BMJ Open Diabetes Res Care 5:e000336. 10.1136/bmjdrc-2016-000336 28761647 10.1136/bmjdrc-2016-000336 PMC5530269

[8] Chávez-Reyes J, Escárcega-González CE, Chavira-Suárez E et al (2021) Susceptibility for some infectious diseases in patients with diabetes: the key role of glycemia. Front Public Health 9. 10.3389/fpubh.2021.559595 10.3389/fpubh.2021.559595 PMC7921169 33665182

[9] Carey IM, Critchley JA, DeWilde S et al (2018) Risk of infection in type 1 and type 2 diabetes compared with the general population: A matched cohort study. Diabetes Care 41:513–521. 10.2337/dc17-2131 29330152 10.2337/dc17-2131

[10] Harding JL, Benoit SR, Gregg EW et al (2020) Trends in rates of infections requiring hospitalization among adults with versus without diabetes in the U.S., 2000–2015. Diabetes Care 43:106–116. 10.2337/dc19-0653 31615853 10.2337/dc19-0653

[11] Berbudi A, Rahmadika N, Tjahjadi AI, Ruslami R (2020) Type 2 diabetes and its impact on the immune system. Curr Diabetes Rev 16:442–449. 10.2174/1573399815666191024085838 31657690 10.2174/1573399815666191024085838 PMC7475801

[12] Ministero della Salute Piano Nazionale Prevenzione Vaccinale (PNPV) 2023–25. https://www.quotidianosanita.it/allegati/allegato1679488094.pdf. Accessed 30 Jul 2024

[13] IDF Europe (2021) Position Paper on Vaccination of People living with Diabetes. https://idf.org/europe/news/idf-europe-position-paper-on-vaccination-of-people-living-with-diabetes/. Accessed 10 Sep 2024

[14] ElSayed NA, Aleppo G, Bannuru RR et al (2024) 4. Comprehensive medical evaluation and assessment of comorbidities: *Standards of care in Diabetes—2024*. Diabetes Care 47:S52–S76. 10.2337/dc24-S004 38078591 10.2337/dc24-S004 PMC10725809

[15] Silvestris N, Franchina T, Gallo M et al (2023) Diabetes management in cancer patients. An Italian association of medical oncology, Italian association of medical diabetologists, Italian society of diabetology, Italian society of endocrinology and Italian society of Pharmacology multidisciplinary consensus position paper. ESMO Open 8:102062. 10.1016/j.esmoop.2023.102062 38070434 10.1016/j.esmoop.2023.102062 PMC10714217

[16] Woodfield MC, Pergam SA, Shah PD (2021) Cocooning against COVID-19: the argument for vaccinating caregivers of patients with cancer. Cancer 127:2861–2863. 10.1002/cncr.33598 33891713 10.1002/cncr.33598 PMC8251451

[17] Freedman M, Kroger A, Hunter P, Ault KA (2020) Recommended adult immunization schedule, united States, 2020*. Ann Intern Med 172:337. 10.7326/M20-0046 32016359 10.7326/M20-0046 PMC9555290

[18] Blonde L, Umpierrez GE, Reddy SS et al (2022) American association of clinical endocrinology clinical practice guideline: developing a diabetes mellitus comprehensive care Plan—2022 update. Endocr Pract 28:923–1049. 10.1016/j.eprac.2022.08.002 35963508 10.1016/j.eprac.2022.08.002 PMC10200071

[19] Icardi G, Francia F, Di Bartolo P et al (2018) Multi-disciplinary consensus statement document vaccinal prevention in adult patients with diabetes mellitus. J Prev Med Hyg 59:E249–E256. 10.15167/2421-4248/jpmh2018.59.4.1124 30656226 10.15167/2421-4248/jpmh2018.59.4.1124 PMC6319117

[20] Rieger CT, Liss B, Mellinghoff S et al (2018) Anti-infective vaccination strategies in patients with hematologic malignancies or solid tumors—Guideline of the infectious diseases working party (AGIHO) of the German society for hematology and medical oncology (DGHO). Ann Oncol 29:1354–1365. 10.1093/annonc/mdy117 29688266 10.1093/annonc/mdy117 PMC6005139

[21] Rubin LG, Levin MJ, Ljungman P et al (2014) Executive summary: 2013 IDSA clinical practice guideline for vaccination of the immunocompromised host. Clin Infect Dis 58:309–318. 10.1093/cid/cit816 24421306 10.1093/cid/cit816

[22] Ministero della Salute Prevenzione e controllo dell’influenza: raccomandazioni per la stagione 2024–2025. https://www.trovanorme.salute.gov.it/norme/renderNormsanPdf?anno=2024&codLeg=100738&parte=1%20&serie=null. Accessed 10 Oct 2024

[23] Ariza-Heredia EJ, Chemaly RF (2015) Practical review of immunizations in adult patients with cancer. Hum Vaccin Immunother 11:2606–2614. 10.1080/21645515.2015.1062189 26110220 10.1080/21645515.2015.1062189 PMC4685676

[24] Rüthrich MM, Giesen N, Mellinghoff SC et al (2022) Cellular immune response after vaccination in patients with Cancer—Review on past and present experiences. Vaccines (Basel) 10:182. 10.3390/vaccines10020182 35214642 10.3390/vaccines10020182 PMC8875094

[25] Chiou W-Y, Hung S-K, Lin H-Y et al (2019) Effectiveness of 23-valent Pneumococcal polysaccharide vaccine on elderly patients with colorectal cancer. Medicine 98:e18380. 10.1097/MD.0000000000018380 31852152 10.1097/MD.0000000000018380 PMC6922596

[26] Piubelli C, Valerio M, Verzè M et al (2023) Humoral effect of SARS-CoV-2 mRNA vaccination with booster dose in solid tumor patients with different anticancer treatments. Front Oncol 13. 10.3389/fonc.2023.1089944 10.3389/fonc.2023.1089944 PMC9992722 36910621

[27] Best AF, Bowman M, Li J et al (2023) COVID-19 severity by vaccination status in the NCI COVID-19 and cancer patients study (NCCAPS). JNCI: J Natl Cancer Inst 115:597–600. 10.1093/jnci/djad015 36702472 10.1093/jnci/djad015 PMC10165483

[28] Choueiri TK, Labaki C, Bakouny Z et al (2023) Breakthrough SARS-CoV-2 infections among patients with cancer following two and three doses of COVID-19 mRNA vaccines: a retrospective observational study from the COVID-19 and cancer consortium. Lancet Reg Health - Americas 19:100445. 10.1016/j.lana.2023.100445 36818595 10.1016/j.lana.2023.100445 PMC9925160

[29] Cortellini A, Tabernero J, Mukherjee U et al (2023) SARS-CoV-2 Omicron (B.1.1.529)-related COVID-19 sequelae in vaccinated and unvaccinated patients with cancer: results from the OnCovid registry. Lancet Oncol 24:335–346. 10.1016/S1470-2045(23)00056-6 36898391 10.1016/S1470-2045(23)00056-6 PMC9991062

[30] Gobbato M, Clagnan E, Toffolutti F et al (2023) Vaccination against SARS-CoV-2 and risk of hospital admission and death among infected cancer patients: A population-based study in Northern Italy. Cancer Epidemiol 82:102318. 10.1016/j.canep.2022.102318 36566579 10.1016/j.canep.2022.102318 PMC9760613

[31] Tagliamento M, Gennari A, Lambertini M et al (2023) Pandemic Phase-Adjusted analysis of COVID-19 outcomes reveals reduced intrinsic vulnerability and substantial vaccine protection from severe acute respiratory syndrome coronavirus 2 in patients with breast cancer. J Clin Oncol 41:2800–2814. 10.1200/JCO.22.01667 36720089 10.1200/JCO.22.01667 PMC10414724

[32] Lau D, Eurich DT, Majumdar SR et al (2014) Working-age adults with diabetes experience greater susceptibility to seasonal influenza: a population-based cohort study. Diabetologia 57:690–698. 10.1007/s00125-013-3158-8 24496923 10.1007/s00125-013-3158-8

[33] Bechini A, Ninci A, Del Riccio M et al (2020) Impact of influenza vaccination on All-Cause mortality and hospitalization for pneumonia in adults and the elderly with diabetes: A Meta-Analysis of observational studies. Vaccines (Basel) 8263. 10.3390/vaccines8020263 10.3390/vaccines8020263 PMC7349976 32486233

[34] Vamos EP, Pape UJ, Curcin V et al (2016) Effectiveness of the influenza vaccine in preventing admission to hospital and death in people with type 2 diabetes. Can Med Assoc J 188:E342–E351. 10.1503/cmaj.151059 27455981 10.1503/cmaj.151059 PMC5047834

[35] SItI-AMD-SID RACCOMANDAZIONI PER LA PROFILASSI VACCINALE NEI SOGGETTI AFFETTI DA DIABETE, MELLITO DI TIPO 1 E 2. https://aemmedi.it/wp-content/uploads/2023/01/RACCOMANDAZIONI_PER_LA_PROFILASSI_VACCINALE.pdf. Accessed 14 Oct 2024

[36] European Union EUR-Lex– 32009H1019– EN. https://eur-lex.europa.eu/eli/reco/2009/1019/oj. Accessed 1 Aug 2024

[37] Istituto Superiore di Sanità EpiCentro - L’epidemiologia per la sanità pubblica. Sorveglianza PASSI. https://www.epicentro.iss.it/passi/dati/VaccinazioneAntinfluenzale. Accessed 31 Jul 2024

[38] Jiménez-Garcia R, Lopez-de-Andres A, Hernandez-Barrera V et al (2017) Influenza vaccination in people with type 2 diabetes, coverage, predictors of uptake, and perceptions. Result of the MADIABETES cohort a 7years follow up study. Vaccine 35:101–108. 10.1016/j.vaccine.2016.11.039 27890398 10.1016/j.vaccine.2016.11.039

[39] Bocquier A, Cortaredona S, Fressard L et al (2019) Trajectories of seasonal influenza vaccine uptake among French people with diabetes: a nationwide retrospective cohort study, 2006–2015. BMC Public Health 19:918. 10.1186/s12889-019-7209-z 31288768 10.1186/s12889-019-7209-z PMC6617633

[40] Papagianni M, Metallidis S, Tziomalos K (2018) Herpes Zoster and diabetes mellitus: A review. Diabetes Therapy 9:545–550. 10.1007/s13300-018-0394-4 29520743 10.1007/s13300-018-0394-4 PMC6104256

[41] Mastrovito B, Lardon A, Dubromel A et al (2024) Understanding the gap between guidelines and influenza vaccination coverage in people with diabetes: a scoping review. Front Public Health 12. 10.3389/fpubh.2024.1360556 10.3389/fpubh.2024.1360556 PMC11066301 38706547

[42] WHO Publication (2012) Pneumococcal vaccines WHO position paper– 2012– Recommendations. Vaccine 30:4717–4718. 10.1016/j.vaccine.2012.04.093 22621828 10.1016/j.vaccine.2012.04.093

[43] Rao Kondapally Seshasai S, Kaptoge S, Thompson A et al (2011) Diabetes mellitus, fasting glucose, and risk of cause-specific death. N Engl J Med 364:829–841. 10.1056/NEJMoa1008862 21366474 10.1056/NEJMoa1008862 PMC4109980

[44] Diepersloot RJA, Bouter KP, Beyer WEP et al (1987) Humoral immune response and delayed type hypersensitivity to influenza vaccine in patients with diabetes mellitus. Diabetologia 30:397–401. 10.1007/BF00292541 3678660 10.1007/BF00292541

[45] Muszkat M, Friedman G, Dannenberg HD et al (2003) Response to influenza vaccination in community and in nursing home residing elderly: relation to clinical factors. Exp Gerontol 38:1199–1203. 10.1016/j.exger.2003.07.004 14580873 10.1016/j.exger.2003.07.004

[46] Pozzilli P, Arduini P, Visalli N et al (1987) Reduced protection against hepatitis B virus following vaccination in patients with type 1 (insulin-dependent) diabetes. Diabetologia 30. 10.1007/BF00275749 10.1007/BF00275749 2962892

[47] El-Madhun AS, Cox RJ, Seime A et al (1998) Systemic and local immune responses after parenteral influenza vaccination in juvenile diabetic patients and healthy controls: results from a pilot study. Vaccine 16:156–160. 10.1016/S0264-410X(97)88328-4 9607024 10.1016/s0264-410x(97)88328-4

[48] Pozzilli P, Gale EAM, Visallil N et al (1986) The immune response to influenza vaccination in diabetic patients. Diabetologia 29:850–854. 10.1007/BF00870139 3569690 10.1007/BF00870139

[49] Dicembrini I, Vitale V, Cosentino C et al (2022) Interstitial glucose monitoring, type 1 diabetes and COVID-19 vaccine: the patient-reported outcomes and vaccine-associated changes in glucose and side effects (PRO-VACS). Acta Diabetol 59:435–438. 10.1007/s00592-021-01837-0 35088165 10.1007/s00592-021-01837-0 PMC8794636

[50] Diao W, Shen N, Yu P et al (2016) Efficacy of 23-valent Pneumococcal polysaccharide vaccine in preventing community-acquired pneumonia among immunocompetent adults: A systematic review and meta-analysis of randomized trials. Vaccine 34:1496–1503. 10.1016/j.vaccine.2016.02.023 26899376 10.1016/j.vaccine.2016.02.023

[51] Farrar JL, Childs L, Ouattara M et al (2023) Systematic review and Meta-Analysis of the efficacy and effectiveness of Pneumococcal vaccines in adults. Pathogens 12:732. 10.3390/pathogens12050732 37242402 10.3390/pathogens12050732 PMC10222197

[52] Huijts SM, van Werkhoven CH, Bolkenbaas M et al (2017) Post-hoc analysis of a randomized controlled trial: diabetes mellitus modifies the efficacy of the 13-valent Pneumococcal conjugate vaccine in elderly. Vaccine 35:4444–4449. 10.1016/j.vaccine.2017.01.071 28410813 10.1016/j.vaccine.2017.01.071

[53] Silverii GA, Gabutti G, Tafuri S et al (2024) Diabetes as a risk factor for Pneumococcal disease and severe related outcomes and efficacy/effectiveness of vaccination in diabetic population. Results from meta-analysis of observational studies. Acta Diabetol 61:1029–1039. 10.1007/s00592-024-02282-5 38684540 10.1007/s00592-024-02282-5 PMC11329702

[54] Verstraeten T, Fletcher MA, Suaya JA et al (2020) Diabetes mellitus as a vaccine-effect modifier: a review. Expert Rev Vaccines 19:445–453. 10.1080/14760584.2020.1760098 32516066 10.1080/14760584.2020.1760098

[55] Del Riccio M, Boccalini S, Cosma C et al (2023) Effectiveness of Pneumococcal vaccination on hospitalization and death in the adult and older adult diabetic population: a systematic review. Expert Rev Vaccines 22:1179–1184. 10.1080/14760584.2023.2286374 37990793 10.1080/14760584.2023.2286374

[56] Kobayashi M, Farrar JL, Gierke R et al (2022) Use of 15-Valent Pneumococcal conjugate vaccine and 20-Valent Pneumococcal conjugate vaccine among U.S. Adults: updated recommendations of the advisory committee on immunization Practices — United States, 2022. MMWR Morb Mortal Wkly Rep 71:109–117. 10.15585/mmwr.mm7104a1 35085226 10.15585/mmwr.mm7104a1 PMC9351524

[57] Remschmidt C, Wichmann O, Harder T (2015) Vaccines for the prevention of seasonal influenza in patients with diabetes: systematic review and meta-analysis. BMC Med 13:53. 10.1186/s12916-015-0295-6 25857236 10.1186/s12916-015-0295-6 PMC4373029

[58] Clar C, Oseni Z, Flowers N et al (2015) Influenza vaccines for preventing cardiovascular disease. Cochrane Database Syst Reviews 2015. 10.1002/14651858.CD005050.pub3 10.1002/14651858.CD005050.pub3 PMC8511741 25940444

[59] Hulme KD, Gallo LA, Short KR (2017) Influenza virus and glycemic variability in diabetes: A killer combination?? Front Microbiol. 10.3389/fmicb.2017.00861. 8:28588558 10.3389/fmicb.2017.00861 PMC5438975

[60] Huang C-T, Lee C-Y, Sung H-Y et al (2022) Association between diabetes mellitus and the risk of herpes Zoster: A systematic review and Meta-analysis. J Clin Endocrinol Metab 107:586–597. 10.1210/clinem/dgab675 34536279 10.1210/clinem/dgab675

[61] Saadatian-Elahi M, Bauduceau B, Del-Signore C, Vanhems P (2020) Diabetes as a risk factor for herpes Zoster in adults: A synthetic literature review. Diabetes Res Clin Pract 159:107983. 10.1016/j.diabres.2019.107983 31846665 10.1016/j.diabres.2019.107983

[62] Silverii GA, Clerico A, Fornengo R et al (2023) Efficacy and effectiveness of herpes Zoster vaccination in adults with diabetes mellitus: a systematic review and meta-analysis of clinical trials and observational studies. Acta Diabetol 60:1343–1349. 10.1007/s00592-023-02127-7 37340183 10.1007/s00592-023-02127-7 PMC10442285

[63] Shi Y, Hu FB (2014) The global implications of diabetes and cancer. Lancet 383:1947–1948. 10.1016/S0140-6736(14)60886-2 24910221 10.1016/S0140-6736(14)60886-2

[64] Wang M, Yang Y, Liao Z (2020) Diabetes and cancer: epidemiological and biological links. World J Diabetes 11:227–238. 10.4239/wjd.v11.i6.227 32547697 10.4239/wjd.v11.i6.227 PMC7284016

[65] Natalicchio A, Marrano N, Montagnani M et al (2024) Glycemic control and cancer outcomes in oncologic patients with diabetes: an Italian association of medical oncology (AIOM), Italian association of medical diabetologists (AMD), Italian society of diabetology (SID), Italian society of endocrinology (SIE), Italian society of Pharmacology (SIF) multidisciplinary critical view. J Endocrinol Invest. 10.1007/s40618-024-02417-z 38935200 10.1007/s40618-024-02417-z PMC11549129

[66] Li S, Wang J, Zhang B et al (2019) Diabetes mellitus and Cause-Specific mortality: A Population-Based study. Diabetes Metab J 43:319. 10.4093/dmj.2018.0060 31210036 10.4093/dmj.2018.0060 PMC6581547

[67] Casqueiro J, Casqueiro J, Alves C (2012) Infections in patients with diabetes mellitus: A review of pathogenesis. Indian J Endocrinol Metab 16:27. 10.4103/2230-8210.94253 10.4103/2230-8210.94253 PMC3354930 22701840

[68] Rammaert B, Lanternier F, Poirée S et al (2012) Diabetes and mucormycosis: A complex interplay. Diabetes Metab 38:193–204. 10.1016/j.diabet.2012.01.002 22386924 10.1016/j.diabet.2012.01.002

[69] Renehan AG, Yeh H-C, Johnson JA et al (2012) Diabetes and cancer (2): evaluating the impact of diabetes on mortality in patients with cancer. Diabetologia 55:1619–1632. 10.1007/s00125-012-2526-0 22476948 10.1007/s00125-012-2526-0

[70] Barone BB (2008) Long-term All-Cause mortality in cancer patients with preexisting diabetes mellitus. JAMA 300:2754. 10.1001/jama.2008.824 19088353 10.1001/jama.2008.824 PMC3093051

[71] Donnelly JP, Blijlevens NMA, De Pauw BE (2009) Infections in the immunocompromised host: general principles. Churchill Livingstone, Philadelphia

[72] Todar KG (2008) Immune defense against bacterial pathogens. adaptive or acquired immunity

[73] Todar KG (2008) Immune defense against bacterial pathogens: innate immunity

[74] Shahid RK, Ahmed S, Le D, Yadav S (2021) Diabetes and cancer: risk, challenges, management and outcomes. Cancers (Basel) 13:5735. 10.3390/cancers13225735 34830886 10.3390/cancers13225735 PMC8616213

[75] Li Q (2023) Bacterial infection and microbiota in carcinogenesis and tumor development. Front Cell Infect Microbiol 13. 10.3389/fcimb.2023.1294082 10.3389/fcimb.2023.1294082 PMC10684967 38035341

[76] Larsson SC, Wolk A (2011) Diabetes mellitus and incidence of kidney cancer: a meta-analysis of cohort studies. Diabetologia 54:1013–1018. 10.1007/s00125-011-2051-6 21274512 10.1007/s00125-011-2051-6

[77] Joung KH, Jeong J-W, Ku BJ (2015) The association between type 2 diabetes mellitus and women cancer: the epidemiological evidences and putative mechanisms. Biomed Res Int 2015:1–12. 10.1155/2015/920618 10.1155/2015/920618 PMC4383430 25866823

[78] Larsson SC, Orsini N, Wolk A (2005) Diabetes mellitus and risk of colorectal cancer: A Meta-Analysis. JNCI: J Natl Cancer Inst 97:1679–1687. 10.1093/jnci/dji375 16288121 10.1093/jnci/dji375

[79] Neale RE, Doecke JD, Pandeya N et al (2009) Does type 2 diabetes influence the risk of oesophageal adenocarcinoma? Br J Cancer 100:795–798. 10.1038/sj.bjc.6604908 19190630 10.1038/sj.bjc.6604908 PMC2653775

[80] Bădulescu F, Stan MC, Crişan A et al (2017) Epidemiological aspects of oncological pathology in patients with diabetes (2007–2017). 10.26416/OnHe.38.1.2017.587. Oncolog-Hematolog.ro 1:

[81] Ling S, Brown K, Miksza JK et al (2020) Association of type 2 diabetes with cancer: A Meta-analysis with bias analysis for unmeasured confounding in 151 cohorts comprising 32 million people. Diabetes Care 43:2313–2322. 10.2337/dc20-0204 32910779 10.2337/dc20-0204

[82] Zhu B, Qu S (2022) The relationship between diabetes mellitus and cancers and its underlying mechanisms. Front Endocrinol (Lausanne) 13. 10.3389/fendo.2022.800995 10.3389/fendo.2022.800995 PMC8873103 35222270

[83] Ramteke P, Deb A, Shepal V, Bhat MK (2019) Hyperglycemia associated metabolic and molecular alterations in cancer risk, progression, treatment, and mortality. Cancers (Basel) 11:1402. 10.3390/cancers11091402 31546918 10.3390/cancers11091402 PMC6770430

[84] Ryu TY, Park J, Scherer PE (2014) Hyperglycemia as a risk factor for cancer progression. Diabetes Metab J 38:330. 10.4093/dmj.2014.38.5.330 25349819 10.4093/dmj.2014.38.5.330 PMC4209346

[85] Nasir Kansestani A, Mansouri K, Hemmati S et al (2019) High Glucose-reduced apoptosis in human breast cancer cells is mediated by activation of NF-κB. Iran J Allergy Asthma Immunol 18:153–162 31066251

[86] Guo J, Ye F, Jiang X et al (2020) Drp1 mediates high glucose-induced mitochondrial dysfunction and epithelial-mesenchymal transition in endometrial cancer cells. Exp Cell Res 389:111880. 10.1016/j.yexcr.2020.111880 32017930 10.1016/j.yexcr.2020.111880

[87] Rahn S, Zimmermann V, Viol F et al (2018) Diabetes as risk factor for pancreatic cancer: hyperglycemia promotes epithelial-mesenchymal-transition and stem cell properties in pancreatic ductal epithelial cells. Cancer Lett 415:129–150. 10.1016/j.canlet.2017.12.004 29222037 10.1016/j.canlet.2017.12.004

[88] Biernacka KM, Uzoh CC, Zeng L et al (2013) Hyperglycaemia-induced chemoresistance of prostate cancer cells due to IGFBP2. Endocr Relat Cancer 20:741–751. 10.1530/ERC-13-0077 23959956 10.1530/ERC-13-0077

[89] Yang I-P, Miao Z-F, Huang C-W et al (2019) High blood sugar levels but not diabetes mellitus significantly enhance oxaliplatin chemoresistance in patients with stage III colorectal cancer receiving adjuvant FOLFOX6 chemotherapy. Ther Adv Med Oncol 11. 10.1177/1758835919866964 10.1177/1758835919866964 PMC6704420 31467597

[90] Leshem Y, Dolev Y, Siegelmann-Danieli N et al (2023) Association between diabetes mellitus and reduced efficacy of pembrolizumab in non–small cell lung cancer. Cancer 129:2789–2797. 10.1002/cncr.34918 37354065 10.1002/cncr.34918

[91] Gregg EW, Sattar N, Ali MK (2016) The changing face of diabetes complications. Lancet Diabetes Endocrinol 4:537–547. 10.1016/S2213-8587(16)30010-9 27156051 10.1016/S2213-8587(16)30010-9

[92] Natalicchio A, Faggiano A, Zatelli MC et al (2022) Metabolic disorders and gastroenteropancreatic-neuroendocrine tumors (GEP-NETs): how do they influence each other? An Italian association of medical oncology (AIOM)/ Italian association of medical diabetologists (AMD)/ Italian society of endocrinology (SIE)/ Italian society of Pharmacology (SIF) multidisciplinary consensus position paper. Crit Rev Oncol Hematol 169:103572. 10.1016/j.critrevonc.2021.103572 34954047 10.1016/j.critrevonc.2021.103572

[93] Hershey DS (2017) Importance of glycemic control in cancer patients with diabetes: treatment through end of life. Asia Pac J Oncol Nurs 4:313–318. 10.4103/apjon.apjon_40_17 28966959 10.4103/apjon.apjon_40_17 PMC5559941

[94] Joharatnam-Hogan N, Chambers P, Dhatariya K, Board R (2022) A guideline for the outpatient management of glycaemic control in people with cancer. Diabet Med 39. 10.1111/dme.14636 10.1111/dme.14636 34240470

[95] Pearson-Stuttard J, Buckley J, Cicek M, Gregg EW (2021) The changing nature of mortality and morbidity in patients with diabetes. Endocrinol Metab Clin North Am 50:357–368. 10.1016/j.ecl.2021.05.001 34399950 10.1016/j.ecl.2021.05.001

[96] Pearson-Stuttard J, Bennett J, Cheng YJ et al (2021) Trends in predominant causes of death in individuals with and without diabetes in England from 2001 to 2018: an epidemiological analysis of linked primary care records. Lancet Diabetes Endocrinol 9:165–173. 10.1016/S2213-8587(20)30431-9 33549162 10.1016/S2213-8587(20)30431-9 PMC7886654

[97] Hwangbo Y, Kang D, Kang M et al (2018) Incidence of diabetes after cancer development. JAMA Oncol 4:1099. 10.1001/jamaoncol.2018.1684 29879271 10.1001/jamaoncol.2018.1684 PMC6143049

[98] Schmidt SF, Rohm M, Herzig S, Berriel Diaz M (2018) Cancer cachexia: more than skeletal muscle wasting. Trends Cancer 4:849–860. 10.1016/j.trecan.2018.10.001 30470306 10.1016/j.trecan.2018.10.001

[99] Chowdhury TA, Jacob P (2019) Challenges in the management of people with diabetes and cancer. Diabet Med 36:795–802. 10.1111/dme.13919 30706527 10.1111/dme.13919

[100] Hershey DS, Tipton J, Given B, Davis E (2012) Perceived impact of cancer treatment on diabetes Self-Management. Diabetes Educ 38:779–790. 10.1177/0145721712458835 22983823 10.1177/0145721712458835

[101] Gallo M, Muscogiuri G, Felicetti F et al (2018) Adverse glycaemic effects of cancer therapy: indications for a rational approach to cancer patients with diabetes. Metabolism 78:141–154. 10.1016/j.metabol.2017.09.013 28993227 10.1016/j.metabol.2017.09.013

[102] Ashley L, Kassim S, Kellar I et al (2022) Identifying ways to improve diabetes management during cancer treatments (INDICATE): protocol for a qualitative interview study with patients and clinicians. BMJ Open 12:e060402. 10.1136/bmjopen-2021-060402 35193924 10.1136/bmjopen-2021-060402 PMC8867345

[103] Hershey DS, Given B, Given C et al (2014) Predictors of diabetes Self-Management in older adults receiving chemotherapy. Cancer Nurs 37:97–105. 10.1097/NCC.0b013e3182888b14 23519039 10.1097/NCC.0b013e3182888b14

[104] Faggiano A, Mazzilli R, Natalicchio A et al (2022) Corticosteroids in oncology: use, overuse, indications, contraindications. An Italian association of medical oncology (AIOM)/ Italian association of medical diabetologists (AMD)/ Italian society of endocrinology (SIE)/ Italian society of Pharmacology (SIF) multidisciplinary consensus position paper. Crit Rev Oncol Hematol 180:103826. 10.1016/j.critrevonc.2022.103826 36191821 10.1016/j.critrevonc.2022.103826

[105] Shariff AI, Syed S, Shelby RA et al (2019) Novel cancer therapies and their association with diabetes. J Mol Endocrinol 62:R187–R199. 10.1530/JME-18-0002 30532995 10.1530/JME-18-0002

[106] Silvestris N, Argentiero A, Beretta GD et al (2020) Management of metabolic adverse events of targeted therapies and immune checkpoint inhibitors in cancer patients: an associazione Italiana oncologia medica (AIOM)/Associazione Medici diabetologi (AMD)/Società Italiana Farmacologia (SIF) multidisciplinary consensus position paper. Crit Rev Oncol Hematol 154:103066. 10.1016/j.critrevonc.2020.103066 32853883 10.1016/j.critrevonc.2020.103066

[107] Alberti KGMM, Juel Christensen N, Engkjær Christensen S et al (1973) INHIBITION OF INSULIN SECRETION BY SOMATOSTATIN. Lancet 302:1299–1301. 10.1016/S0140-6736(73)92873-0 10.1016/s0140-6736(73)92873-0 4127645

[108] Hansen L, Hartmann B, Bisgaard T et al (2000) Somatostatin restrains the secretion of glucagon-like peptide-1 and– 2 from isolated perfused Porcine ileum. Am J Physiology-Endocrinology Metabolism 278:E1010–E1018. 10.1152/ajpendo.2000.278.6.E1010 10.1152/ajpendo.2000.278.6.E1010 10827002

[109] Zheng Z, Liu Y, Yang J et al (2021) Diabetes mellitus induced by immune checkpoint inhibitors. Diabetes Metab Res Rev 37. 10.1002/dmrr.3366 10.1002/dmrr.3366 32543027

[110] Loubet P, Wittkop L, Ninove L et al (2023) One-month humoral response following two or three doses of messenger RNA coronavirus disease 2019 vaccines as primary vaccination in specific populations in France: first results from the agence Nationale recherche Contre Le Sida (ANRS)0001S COV-POPART cohort. Clin Microbiol Infect 29:388e1. 10.1016/j.cmi.2022.10.009 10.1016/j.cmi.2022.10.009 PMC9562615 36252789

[111] Porntharukchareon T, Chartisathian W, Navinpipat M et al (2023) The immunogenicity of the ChAdOx1 nCoV-19 vaccination in participants with underlying comorbidity diseases: A prospective cohort study. Hum Vaccin Immunother 19. 10.1080/21645515.2023.2251850 10.1080/21645515.2023.2251850 PMC10484043 37671943

[112] Zheng J-Q, Lin C-H, Chen C-C et al (2021) Role of annual influenza vaccination against lung cancer in type 2 diabetic patients from a Population-Based cohort study. J Clin Med 10:3434. 10.3390/jcm10153434 34362218 10.3390/jcm10153434 PMC8347140

[113] Newman JH, Chesson CB, Herzog NL et al (2020) Intratumoral injection of the seasonal flu shot converts immunologically cold tumors to hot and serves as an immunotherapy for cancer. Proceedings of the National Academy of Sciences 117:1119–1128. 10.1073/pnas.1904022116 10.1073/pnas.1904022116 PMC6969546 31888983

[114] Nojima I, Eikawa S, Tomonobu N et al (2020) Dysfunction of CD8 + PD-1 + T cells in type 2 diabetes caused by the impairment of metabolism-immune axis. Sci Rep 10:14928. 10.1038/s41598-020-71946-3 32913271 10.1038/s41598-020-71946-3 PMC7484782

[115] Saleh R, Sasidharan Nair V, Murshed K et al (2021) Transcriptome of CD8 + tumor-infiltrating T cells: a link between diabetes and colorectal cancer. Cancer Immunol Immunother 70:2625–2638. 10.1007/s00262-021-02879-7 33582867 10.1007/s00262-021-02879-7 PMC10992610

[116] Lasagna A, Brunello A, Silvestris N et al (2024) Italian oncologists and vaccinations against infectious diseases: results of a survey of the Italian association of medical oncology. Tumori J 110:60–68. 10.1177/03008916231191547 10.1177/03008916231191547 PMC10851644 37586016

[117] Parekh AK, Goodman RA, Gordon C, Koh HK (2011) Managing multiple chronic conditions: A strategic framework for improving health outcomes and quality of life. Public Health Reports^®^ 126:460–471. 10.1177/003335491112600403 21800741 10.1177/003335491112600403 PMC3115206

[118] Pedrazzoli P, Piralla A, Valentino F et al (2018) Update of the recommendations of the Italian society of medical oncology on vaccination for seasonal influenza and Pneumococcal infection in patients with cancer: focus on prevention of pneumonia. Eur J Cancer Care (Engl) 27:e12817. 10.1111/ecc.12817 29575267 10.1111/ecc.12817

[119] Pedrazzoli P, Lasagna A, Cassaniti I et al (2022) Vaccination for herpes Zoster in patients with solid tumors: a position paper on the behalf of the associazione Italiana Di oncologia medica (AIOM). ESMO Open 7:100548. 10.1016/j.esmoop.2022.100548 35853350 10.1016/j.esmoop.2022.100548 PMC9434335

[120] Zheng Y, Chen Y, Yu K et al (2021) Fatal infections among cancer patients: A Population-Based study in the united States. Infect Dis Ther 10:871–895. 10.1007/s40121-021-00433-7 33761114 10.1007/s40121-021-00433-7 PMC8116465

[121] Ayoola A, Sukumaran S, Jain K et al (2020) Efficacy of influenza vaccine (Fluvax) in cancer patients on treatment: a prospective single arm, open-label study. Support Care Cancer 28:5411–5417. 10.1007/s00520-020-05384-2 32144585 10.1007/s00520-020-05384-2

[122] Strowd RE, Swett K, Harmon M et al (2014) Influenza vaccine immunogenicity in patients with primary central nervous system malignancy. Neuro Oncol 16:1639–1644. 10.1093/neuonc/nou051 24714522 10.1093/neuonc/nou051 PMC4232079

[123] DiazGranados CA, Dunning AJ, Kimmel M et al (2014) Efficacy of High-Dose versus Standard-Dose influenza vaccine in older adults. N Engl J Med 371:635–645. 10.1056/NEJMoa1315727 25119609 10.1056/NEJMoa1315727

[124] Grohskopf LA, Blanton LH, Ferdinands JM et al (2022) Prevention and control of seasonal influenza with vaccines: recommendations of the advisory committee on immunization Practices — United States, 2022–23 influenza season. MMWR Recommendations Rep 71:1–28. 10.15585/mmwr.rr7101a1 10.15585/mmwr.rr7101a1 PMC9429824 36006864

[125] Dai M, Liu D, Liu M et al (2020) Patients with cancer appear more vulnerable to SARS-CoV-2: A multicenter study during the COVID-19 outbreak. Cancer Discov 10:783–791. 10.1158/2159-8290.CD-20-0422 32345594 10.1158/2159-8290.CD-20-0422 PMC7309152

[126] Pinato DJ, Zambelli A, Aguilar-Company J et al (2020) Clinical portrait of the SARS-CoV-2 epidemic in European patients with cancer. Cancer Discov 10:1465–1474. 10.1158/2159-8290.CD-20-0773 32737082 10.1158/2159-8290.CD-20-0773 PMC7668225

[127] Mehrabi Nejad M-M, Moosaie F, Dehghanbanadaki H et al (2022) Immunogenicity of COVID-19 mRNA vaccines in immunocompromised patients: a systematic review and meta-analysis. Eur J Med Res 27:23. 10.1186/s40001-022-00648-5 35151362 10.1186/s40001-022-00648-5 PMC8840778

[128] Becerril-Gaitan A, Vaca-Cartagena BF, Ferrigno AS et al (2022) Immunogenicity and risk of severe acute respiratory syndrome coronavirus 2 (SARS-CoV-2) infection after coronavirus disease 2019 (COVID-19) vaccination in patients with cancer: a systematic review and meta-analysis. Eur J Cancer 160:243–260. 10.1016/j.ejca.2021.10.014 34794855 10.1016/j.ejca.2021.10.014 PMC8548030

[129] Center for Disease Control and Prevention Vaccines & Immunizations Use of COVID-19 Vaccines in the United States. Interim Clinical Considerations. https://www.cdc.gov/vaccines/covid-19/clinical-considerations/covid-19-vaccines-us.html. Accessed 28 Apr 2024

[130] Lasagna A, Bergami F, Lilleri D et al (2022) Six-month humoral and cellular immune response to the third dose of BNT162b2 anti-SARS-CoV-2 vaccine in patients with solid tumors: a longitudinal cohort study with a focus on the variants of concern. ESMO Open 7:100574. 10.1016/j.esmoop.2022.100574 36029652 10.1016/j.esmoop.2022.100574 PMC9353611

[131] Di Cosimo S, Lupo-Stanghellini MT, Costantini M et al (2022) Safety of third dose of COVID-19 vaccination in frail patients: results from the prospective Italian VAX4FRAIL study. Front Oncol 12. 10.3389/fonc.2022.1002168 10.3389/fonc.2022.1002168 PMC9631315 36338743

[132] Society of Breast Imaging Patient Care and Delivery Committee Revised SBI Recommendations for the Management of Axillary Adenopathy in Patients With Recent COVID -19 Vaccination. https://www.sbi-online.org/sbi-recommendations-position-statements. Accessed 28 Apr 2024

[133] Brooks LRK, Mias GI (2018) Streptococcus pneumoniae’s virulence and host immunity: aging, diagnostics, and prevention. Front Immunol 9. 10.3389/fimmu.2018.01366 10.3389/fimmu.2018.01366 PMC6023974 29988379

[134] Li L, Ma J, Yu Z et al (2023) Epidemiological characteristics and antibiotic resistance mechanisms of Streptococcus pneumoniae: an updated review. Microbiol Res 266:127221. 10.1016/j.micres.2022.127221 36244081 10.1016/j.micres.2022.127221

[135] Choi W, Kim JG, Beom S-H et al (2020) Immunogenicity and optimal timing of 13-Valent Pneumococcal conjugate vaccination during adjuvant chemotherapy in gastric and colorectal cancer: A randomized controlled trial. Cancer Res Treat 52:246–253. 10.4143/crt.2019.189 31291710 10.4143/crt.2019.189 PMC6962463

[136] Li C, Chen L, Lin H et al (2021) Impact of 23-valent Pneumococcal polysaccharide vaccination on the frequency of pneumonia‐related hospitalization and survival in elderly patients with prostate cancer: A seven‐year nationwide matched cohort study. Cancer 127:124–136. 10.1002/cncr.33203 32997342 10.1002/cncr.33203

[137] Gershon AA, Breuer J, Cohen JI et al (2015) Varicella Zoster virus infection. Nat Rev Dis Primers 1:15016. 10.1038/nrdp.2015.16 27188665 10.1038/nrdp.2015.16 PMC5381807

[138] GlaxoSmithKline Biologicals SHINGRIX https://www.fda.gov/vaccines-blood-biologics/vaccines/shingrix. Accessed 28 Apr 2024

[139] Vink P, Delgado Mingorance I, Maximiano Alonso C et al (2019) Immunogenicity and safety of the adjuvanted Recombinant Zoster vaccine in patients with solid tumors, vaccinated before or during chemotherapy: A randomized trial. Cancer 125:1301–1312. 10.1002/cncr.31909 30707761 10.1002/cncr.31909 PMC6766894

[140] Lasagna A, Mele D, Bergami F et al (2023) The immunogenicity and the safety of the adjuvanted glycoprotein E (gE)-based Recombinant vaccine against herpes Zoster (RZV) in cancer patients during immunotherapy. Hum Vaccin Immunother 19. 10.1080/21645515.2023.2288282 10.1080/21645515.2023.2288282 PMC10732600 38037900

[141] Sun B-W, Zhang P-P, Wang Z-H et al (2024) Prevention and potential treatment strategies for respiratory syncytial virus. Molecules 29:598. 10.3390/molecules29030598 38338343 10.3390/molecules29030598 PMC10856762

[142] Hwang JP, Feld JJ, Hammond SP et al (2020) Hepatitis B virus screening and management for patients with cancer prior to therapy: ASCO provisional clinical opinion update. J Clin Oncol 38:3698–3715. 10.1200/JCO.20.01757 32716741 10.1200/JCO.20.01757 PMC11828660

[143] Weng MK, Doshani M, Khan MA et al (2022) Universal hepatitis B vaccination in adults aged 19–59 years: updated recommendations of the advisory committee on immunization practices—United States, 2022. Am J Transplant 22:1714–1720. 10.1111/ajt.16661 35674154 10.1111/ajt.16661

[144] Ministero della Salute Mpox - Vaccino https://www.salute.gov.it/portale/dispositiviMedici/dettaglioContenutiDispositiviMedici.jsp?id=5911%26area=dispositivi-medici%26menu=vuoto. Accessed 30 Aug 2024

[145] Pool V, Tomovici A, Johnson DR et al (2018) Humoral immunity 10 years after booster immunization with an adolescent and adult formulation combined tetanus, diphtheria, and 5-component acellular pertussis vaccine in the USA. Vaccine 36:2282–2287. 10.1016/j.vaccine.2018.03.029 29573876 10.1016/j.vaccine.2018.03.029

[146] Kamolratanakul S, Pitisuttithum P (2021) Human papillomavirus vaccine efficacy and effectiveness against cancer. Vaccines (Basel) 9:1413. 10.3390/vaccines9121413 34960159 10.3390/vaccines9121413 PMC8706722

[147] Istituto Superiore di Sanità Vaccini disponibili contro l’Hpv. https://www.epicentro.iss.it/hpv/Vaccini-Disponibili. Accessed 30 Aug 2024

[148] Luk AOY, Wu H, Lau ESH et al (2021) Temporal trends in rates of infection-related hospitalisations in Hong Kong people with and without diabetes, 2001–2016: a retrospective study. Diabetologia 64:109–118. 10.1007/s00125-020-05286-2 32986145 10.1007/s00125-020-05286-2 PMC7520551

[149] Shah BR, Hux JE (2003) Quantifying the risk of infectious diseases for people with diabetes. Diabetes Care 26:510–513. 10.2337/diacare.26.2.510 12547890 10.2337/diacare.26.2.510

[150] Khunti K, Valabhji J, Misra S (2023) Diabetes and the COVID-19 pandemic. Diabetologia 66:255–266. 10.1007/s00125-022-05833-z 36418578 10.1007/s00125-022-05833-z PMC9685151

[151] Goeijenbier M, van Sloten TT, Slobbe L et al (2017) Benefits of flu vaccination for persons with diabetes mellitus: A review. Vaccine 35:5095–5101. 10.1016/j.vaccine.2017.07.095 28807608 10.1016/j.vaccine.2017.07.095

[152] Chudasama YV, Zaccardi F, Gillies CL et al (2021) Patterns of Multimorbidity and risk of severe SARS-CoV-2 infection: an observational study in the U.K. BMC Infect Dis 21:908. 10.1186/s12879-021-06600-y 34481456 10.1186/s12879-021-06600-y PMC8418288

[153] Bajpeyi S, Mossayebi A, Kreit H et al (2022) Unmanaged diabetes and elevated blood glucose are poor prognostic factors in the severity and recovery time in predominantly Hispanic hospitalized COVID-19 patients. Front Endocrinol (Lausanne) 13. 10.3389/fendo.2022.861385 10.3389/fendo.2022.861385 PMC9309175 35898451

[154] Kornum JB, Thomsen RW, Riis A et al (2008) Diabetes, glycemic control, and risk of hospitalization with pneumonia. Diabetes Care 31:1541–1545. 10.2337/dc08-0138 18487479 10.2337/dc08-0138 PMC2494631

[155] Suaya JA, Jiang Q, Scott DA et al (2018) Post hoc analysis of the efficacy of the 13-valent Pneumococcal conjugate vaccine against vaccine-type community-acquired pneumonia in at-risk older adults. Vaccine 36:1477–1483. 10.1016/j.vaccine.2018.01.049 29429807 10.1016/j.vaccine.2018.01.049

[156] Kuo C-S, Lu C-W, Chang Y-K et al (2016) Effectiveness of 23-valent Pneumococcal polysaccharide vaccine on diabetic elderly. Medicine 95:e4064. 10.1097/MD.0000000000004064 27368047 10.1097/MD.0000000000004064 PMC4937961

[157] Center for Disease Control and Prevention Pneumococcal Vaccine Recommendations https://www.cdc.gov/pneumococcal/hcp/vaccine-recommendations/. Accessed 10 Jun 2024

[158] Kobayashi M, Pilishvili T, Farrar JL et al (2023) Pneumococcal vaccine for adults aged ≥ 19 years: recommendations of the advisory committee on immunization practices, united States, 2023. MMWR Recommendations Rep 72:1–39. 10.15585/mmwr.rr7203a1 10.15585/mmwr.rr7203a1 PMC10495181 37669242

[159] Mbinta JF, Nguyen BP, Awuni PMA et al (2022) Post-licensure Zoster vaccine effectiveness against herpes Zoster and postherpetic neuralgia in older adults: a systematic review and meta-analysis. Lancet Healthy Longev 3:e263–e275. 10.1016/S2666-7568(22)00039-3 36098300 10.1016/S2666-7568(22)00039-3

[160] Center for Disease Control and Prevention Clinical Considerations for Use of Recombinant Zoster Vaccine (RZV Shingrix) in Immunocompromised Adults Aged ≥ 19 Years. https://www.cdc.gov/shingles/vaccination/immunocompromised-adults.html. Accessed 14 Oct 2024

[161] Redondo E, Rivero-Calle I, Mascarós E et al (2024) Respiratory syncytial virus vaccination recommendations for adults aged 60 years and older: the NeumoExperts prevention group position paper. Arch Bronconeumol 60:161–170. 10.1016/j.arbres.2024.01.004 38311509 10.1016/j.arbres.2024.01.004

[162] Melgar M, Britton A, Roper LE et al (2023) Use of respiratory syncytial virus vaccines in older adults: recommendations of the advisory committee on immunization Practices — United States, 2023. MMWR Morb Mortal Wkly Rep 72:793–801. 10.15585/mmwr.mm7229a4 37471262 10.15585/mmwr.mm7229a4 PMC10360650

[163] Havers FP, Moro PL, Hunter P et al (2020) Use of tetanus toxoid, reduced diphtheria toxoid, and acellular pertussis vaccines: updated recommendations of the advisory committee on immunization Practices — United States, 2019. MMWR Morb Mortal Wkly Rep 69:77–83. 10.15585/mmwr.mm6903a5 31971933 10.15585/mmwr.mm6903a5 PMC7367039

[164] AMD (2018)

[165] Centers for Disease Control and Prevention (CDC) (2011) Use of hepatitis B vaccination for adults with diabetes mellitus: recommendations of the advisory committee on immunization practices (ACIP). MMWR Morb Mortal Wkly Rep 60:1709–1711 22189894

[166] Kohl T, Hamilton A (2018) Evidence-Based Practice Summary Hepatitis B Vaccination in Adults with Diabetes Mellitus

[167] Meites E, Szilagyi PG, Chesson HW et al (2019) Human papillomavirus vaccination for adults: updated recommendations of the advisory committee on immunization practices. MMWR Morb Mortal Wkly Rep 68:698–702. 10.15585/mmwr.mm6832a3 31415491 10.15585/mmwr.mm6832a3 PMC6818701

[168] Alhamar G, Briganti S, Maggi D et al (2023) Prevaccination glucose time in range correlates with antibody response to SARS-CoV-2 vaccine in type 1 diabetes. J Clin Endocrinol Metab 108:e474–e479. 10.1210/clinem/dgad001 36611249 10.1210/clinem/dgad001 PMC10807908

