# Supplementary material for: Vaccination of people with solid tumors and diabetes: existing evidence and recommendations. A position statement from a multidisciplinary panel of scientific societies
Source: J Endocrinol Invest. 2025 Apr 23;48(8):1717–38. doi: 10.1007/s40618-025-02586-5 (PMC12313787; doi:10.1007/s40618-025-02586-5)
Supplement: Supplementary file 1 — Supplementary Material 1 [file 40618_2025_2586_MOESM1_ESM.docx]

Uberto Pagotto, MD, PhD

Editor-in-Chief

Journal of Endocrinological Investigation

Alessandria, February 9^th^, 2025

Dear Prof. Pagotto,

We are glad to resubmit our invited Consensus Statement entitled “*Vaccination of people with solid tumors and diabetes: existing evidence and recommendations. a position statement from a multidisciplinary panel of scientific societies”,* on behalf of a multidisciplinary panel from five Italian scientific societies (AIOM, AMD, SID, SIE, and SIF), to be evaluated for publication in the Journal of Endocrinological Investigation.

We have carefully considered the Referees’ requests and carried out the corrections accordingly: any change to the manuscript is highlighted in yellow. A new figure has been added, as well as a new reference. Please find below, in the text, the details of the revisions to the manuscript and our responses to the referees’ comments point by point, as requested.

Kind regards,

Marco Gallo, MD

(Corresponding author)

**Manuscript ID:** JENI-D-24-01833

**Type of manuscript:** Consensus Statement

**Title:** Vaccination of people with solid tumors and diabetes: existing evidence and recommendations. a position statement from a multidisciplinary panel of scientific societies.

**Authors:** Marco Gallo, Angioletta Lasagna, Valerio Renzelli, Lelio Morviducci, Alessio Cortellini, Matteo Monami, Giampiero Marino, Stefania Gori, Matteo Verzé, Alberto Ragni, Enzo Tuveri, Laura Sciacca, Stella D’Oronzo, Dario Giuffrida, Annalisa Natalicchio, Francesco Giorgino, Nicola Marrano, Maria Chiara Zatelli, Monica Montagnani, Francesco Felicetti, Rossella Mazzilli, Stefano Fogli, Tindara Franchina, Antonella Argentiero, Riccardo Candido, Francesco Perrone, Gianluca Aimaretti, Angelo Avogaro, Nicola Silvestris, Antongiulio Faggiano

**Reviewer #1**

This is a comprehensive position statement for cancer patients with diabetes. An additional figure with an algorithm with a proposal of the timing with sequential or combined visit of the patient to the diabetic doctor and the oncologist to have an optimal diabetic control before the vaccination may be useful to circulate in the medical communities. **Thank you for your suggestion, a new figure with a tentative decision-making algorithm for vaccinating cancer patients with diabetes has been added to the manuscript.**

Some additional minor comments:

Lines 439-440 & 449, please use altogether the characteristics of innate and adaptive immune systems and then use one only definition, i.e. 'into the innate (general or non-specific) compartment and the adaptive (specialized or specific) one [71-73].' and 'Both the innate (general) and the adaptive (specialized) immune systems may be altered in cancer patients. **We have modified the text according to your suggestions.**

Line 504: please add mTOR inhibitor with any corresponding reference (as for example doi: 10.1016/j.tem.2019.11.003) **Thank you, we have modified the text and added a couple of pertinent references.**

**Reviewer #2**

This is an interesting manuscript and I congratulate the authors for their nice collaborative work. **Thank you.** It would be helpful to develop an algorithm to assist multidisciplinary care. **A new figure with a tentative decision-making algorithm for vaccinating cancer patients with diabetes has been added to the manuscript.**

**Reviewer #3**

The manuscript brings together expertise from various disciplines, including oncology, diabetology, and pharmacology, to provide robust recommendations for the unique challenges of vaccinating individuals with cancer and diabetes. This multidisciplinary approach adds significant credibility and a well-rounded perspective. The manuscript presents clear recommendations for healthcare providers, such as vaccine scheduling, specific vaccine types, and strategies to address vaccine hesitancy in this vulnerable population. These actionable items can directly improve patient outcomes. **Thank you for your kind words.**

A more detailed and focused discussion on gaps in current evidence and areas requiring future research would strengthen the manuscript. eg. Common reasons for vaccine hesitancy in these populations (e.g., concerns about immune response, fear of interactions with cancer treatments). Evidence-based strategies to improve vaccination uptake, such as targeted education campaigns or multidisciplinary collaboration between oncologists and diabetologists. **We agree with your suggestions, thank you. We have modified the paragraph “STRATEGIES FOR OVERCOMING CHALLENGES TO VACCINATION IN PATIENTS WITH CANCER AND DIABETES” according to your indications, with some more details and some suggestions for implementing effective strategies.**
